# Canada’s Physical Literacy Consensus Statement: process and outcome

**DOI:** 10.1186/s12889-018-5903-x

**Published:** 2018-10-02

**Authors:** Mark S. Tremblay, Christa Costas-Bradstreet, Joel D. Barnes, Brett Bartlett, Diana Dampier, Chantal Lalonde, Reg Leidl, Patricia Longmuir, Melanie McKee, Rhonda Patton, Richard Way, Jennifer Yessis

**Affiliations:** 1 Healthy Active Living and Obesity Research Group, Children’s Hospital of Eastern Ontario Research Institute, 401 Smyth Road, Ottawa, ON K1H 8L1 Canada; 2 CCB Consulting, Burlington, ON L7L 3A3 Canada; 3 ParticipACTION, Toronto, ON M5S 1M2 Canada; 4 Eastern Ontario Health Unit/Ontario Society of Physical Activity Promoters in Public Health, Cornwall, ON K6H 7E9 Canada; 5 Physical and Health Education Canada, K1H 7X7, Ottawa, ON Canada; 6 Stranmillis University College, Belfast, BT9 5DY UK; 7 Propel Centre for Population Health Impact, University of Waterloo, Waterloo, ON N2L 3G1 Canada; 8 Sport for Life Society, Victoria, BC V8P 3S1 Canada

**Keywords:** Physical literacy, Definition, Consensus, Health, Sedentary behaviour, Physical activity, Motivation, Confidence, Physical competence

## Abstract

**Background:**

Healthy movement behaviours of Canadian children and youth have been found to be suboptimal; this is associated with declines in physical fitness, increases in obesity, and elevated chronic disease risk. Physical literacy is an evolving construct representing foundational domains upon which physically active lifestyles are based. Many sectors and organizations in Canada are embracing physical literacy in their programs, practices, policies, and research; however, the use of inconsistent definitions and conceptualizations of physical literacy had been identified by stakeholders as hindering promotion and advancement efforts.

**Methods:**

With leadership from ParticipACTION, organizations from the physical activity, public health, sport, physical education, and recreation sectors collaborated to create a physical literacy consensus definition and position statement for use by all Canadian organizations and individuals. The process involved an environmental scan, survey of related evidence, stakeholder consultations, and creation of a Steering Committee. From this background work a consensus statement was drafted, shared with stakeholders, revised, and ratified.

**Results:**

*Canada’s Physical Literacy Consensus Statement* was launched in June 2015 at the International Physical Literacy Conference in Vancouver, British Columbia. To further promote the Consensus Statement, the Sport for Life Society developed and simultaneously released the “Vancouver Declaration”, which contained additional guidance on physical literacy. Both the Consensus Statement and the Declaration endorsed the International Physical Literacy Association’s definition of physical literacy, namely “the motivation, confidence, physical competence, knowledge and understanding to value and take responsibility for engagement in physical activities for life”.

**Conclusions:**

Sector partners hope that the Consensus Statement, with its standardized definition, brings greater harmony, synergy, and consistency to physical literacy efforts in Canada and internationally. Going forward, the impact of this initiative on the sector, and the more distal goal of increasing habitual physical activity levels, should be assessed.

**Electronic supplementary material:**

The online version of this article (10.1186/s12889-018-5903-x) contains supplementary material, which is available to authorized users.

## Background

There are global concerns over the current lifestyle behaviours and future health of children and youth [1–8], with similar concerns expressed in Canada [9–13]. Several Canadian initiatives have emerged in recent years in an attempt to mitigate these concerns [13–16]. Recently updated evidence-informed Canadian 24-Hour Movement Guidelines for Children and Youth recommend that for healthy growth and development, children and youth (aged 5–17 years) should achieve high levels of physical activity, low levels of sedentary behaviour, and sufficient sleep each day [13]. A healthy 24 h includes ensuring adequate sleep and reductions in sedentary activities, along with an accumulation of at least 60 min per day of moderate to vigorous physical activity (MVPA) involving a variety of aerobic activities; vigorous physical activities and muscle and bone strengthening activities at least 3 days per week; and several hours of a variety of structured and unstructured light physical activities.

Objectively measured physical activity data show that only 9% of Canadian children and youth aged 5–17 years are getting enough physical activity to meet the guidelines of at least 60 min of MVPA daily [17]. More specifically, 14% of 5- to 11-year olds and 5% of 12- to 17-year olds met the Guidelines on at least 6 days a week; by gender, 6 and 13% of Canadian girls and boys, respectively, met the Guidelines [17]. Among younger children (aged 3–4 years), 70% amassed a minimum of 180 min of light or moderate- to vigorous-intensity physical activity daily [17], consistent with the Canadian Physical Activity Guidelines for the Early Years [18].

In terms of sedentary behaviour, Canadian 5- to 17-year olds spent an average of 8 h and 27 min – nearly two-thirds (or 64%) of their waking hours each day – being sedentary. Those aged 12–17 years were more sedentary than those aged 5–11 years (9 h and 16 min of waking time vs 7 h and 38 min, respectively) while children aged 3–4 years were the least sedentary, at 7 h and 28 min of waking time [17]. New analyses indicate that only 9.5% of Canadian children and youth [19] are meeting the new Canadian 24-Hour Movement Guidelines for Children and Youth [13].

Current habitual movement behaviours of Canadian children and youth warrant concern given the important physical, psycho-social, and academic benefits that are accrued through healthy daily movement [1, 7, 20, 21]. There is unequivocal evidence that healthy physical activity behaviours are foundational for combating non-communicable diseases, including heart disease, stroke, hypertension, osteoporosis, type 2 diabetes, and some cancers among adults [4, 22]. Research suggests that individuals with unhealthy movement behaviours during childhood are more likely to continue these behaviours into adulthood and suffer related adverse health outcomes as adults [23, 24]. There is strong evidence of a global decline in cardiorespiratory fitness in children and adolescents since 1975 [25]. In Canada, there has been a substantial decrease in the overall fitness of Canadian children and youth since 1981 [26], concurrent with unprecedented levels of overweight and obesity [12]. These findings provide compelling evidence to suggest that a significant increase in premature health problems can be anticipated if the physical inactivity crisis is not addressed [27].

### Physical literacy

Given the seminal role physical activity plays in promoting health, it is important to recognize the important contributors to helping Canadians of all ages, backgrounds, circumstances, and abilities become and remain physically active. One important contributor to lifelong physical activity is physical literacy, a relatively new construct first proposed by Whitehead in 1993 [28]. Whitehead conceived physical literacy to encompass the knowledge, skills, and motivation that an individual utilizes to support a physically active lifestyle across the lifespan, and she has continued to champion the promotion, uptake, and interpretation of the construct [29–31]. In particular, she has been a strong proponent of foundational philosophical tenets related to physical literacy [29, 30, 32, 33]. This foundational work, and that of other early leaders, provided the impetus for the formation of the International Physical Literacy Association (https://www.physical-literacy.org.uk/) (IPLA) and a global movement embracing the construct of physical literacy.

Over the past generation, physical literacy has progressively gained momentum as a core construct of physical education, sport, physical activity, recreation, and public health, and it has recently been shown to be positively related to guideline adherence for physical activity and sedentary behaviour [34] as well as cardiorespiratory fitness [35] in a large sample of Canadian children aged 8–12 years. A 2015 report from The Aspen Institute [36] provided an environmental scan on global developments in physical literacy and showed substantial activity in many countries, especially Canada. Review papers [32, 37], commentaries [38], debates [39, 40], and implications for policy [41] have all recently emerged.

Many sectors in Canada, including sport, recreation, physical activity, education, and public health, have embraced physical literacy and are making it a core priority of their business. Notable among these are the formal adoption of physical literacy by the Sport for Life Society (S4L; sportforlife.ca & physicalliteracy.ca), Physical Literacy for Life (physicalliteracyforlife.org), Physical & Health Education Canada (PHE Canada; https://phecanada.ca/activate/physical-literacy), and the Ontario Society of Physical Activity Promoters in Public Health (OSPAPPH; papromoters.blogspot.com). These actions have prompted the development of multiple programs and resources in Canada, and provided an opportunity to establish new partnerships across sectors (e.g., sport and public health); however, this same enthusiasm also created professional friction among organizations as they each established their own leadership role in the area of physical literacy. Exacerbating this tension, and a potential barrier to desired progress, was the multitude of definitions and conceptualizations of physical literacy, and consequent confusion [32, 38]. This confusion was perpetuated in Canada by the development of four assessment tools for physical literacy, each anchored in different definitions: Passport for Life by PHE Canada (passportforlife.ca); Physical Literacy Assessment for Youth (PLAY tools) by S4L (physicalliteracy.ca/play-tools/); Canadian Assessment of Physical Literacy (CAPL; https://www.capl-eclp.ca) by the Healthy Active Living and Obesity Research Group (HALO) at the Children’s Hospital of Eastern Ontario Research Institute; and The Fundamental Movement Skills Assessment Tool by the 60 Minute Kids Club (https://60minkidsclub.org/about/teachers/).

The Aspen Institute report also revealed that each country included in their research had developed their own definition of physical literacy. At the consultative stage, Whitehead advised that a globally embraced definition would be preferable, but understood groups and countries might prefer to reflect their country’s distinctive culture in their definition. According to Whitehead, “If alternative definitions are used, they must identify the core long-term goal of physical literacy as being lifelong participation … and they must make reference to the affective (motivation, confidence, valuing/responsibility), the physical (effective interaction in different contexts) and the cognitive (knowledge and understanding)” [36].

Three organizations providing leadership in the Canadian physical literacy movement – S4L, PHE Canada, and HALO – all had different definitions of physical literacy even though all specified that physical literacy comprised four essential elements: motivation and confidence; physical competence; knowledge and understanding; and engagement in physical activities for life. Despite having common elements, multiple competing definitions were problematic for some local, provincial/territorial, and national organizations that were creating programs, resources, and campaigns. The development and implementation of different evaluation tools to measure physical literacy among children and youth, as described above, also contributed to the challenges.

In 2014, an opportunity to create a common definition emerged through two different but parallel processes. First, inspired by a series of conversations with Dr. Charles Corbin and Dr. Margaret Whitehead, researchers at HALO determined the need for a systematic review of research, a forum for debate, and a process to reach consensus on key issues in physical literacy, including terminology, measurement, and a conceptual model. A comprehensive approach was envisioned whereby researchers in the field could develop a series of background papers to directly address divergent views and existing controversies, and to support discussion. It was also agreed that a consensus meeting was required to connect leading researchers and key experts to review the findings from the background papers and participate in a facilitated discussion, all of which would lead to the development of a consensus statement or position paper that could then be launched at a strategic time.

Concurrently, ParticipACTION (https://www.participaction.com) received a multi-year investment from the Royal Bank of Canada (RBC) and the Public Health Agency of Canada (PHAC) to develop the RBC Learn to Play Project*,* a multipronged effort to enhance physical literacy in children and youth with a goal of encouraging more kids to get outside and play [13]. One of the key strategies of the RBC Learn to Play Project was focused on sector engagement. During the planning phase for this key strategy, the need for a common definition of physical literacy was identified as a way to harmonize the efforts of the growing number of organizations interested in supporting physical literacy at the local, provincial/territorial, and national levels. Given that a common definition had, thus far, proved elusive (possibly due to the limited capacity of the sector to take on such a coordinated effort), it was felt developing such a definition would not only support the implementation of the RBC Learn to Play Project but would also make a significant and lasting contribution. Given the mutual interest in developing a common definition, ParticipACTION and HALO collaborated to orchestrate the development of a common definition of physical literacy and consensus statement.

### Purpose

The purpose of this paper is to describe the processes employed to develop and release *Canada’s Physical Literacy Consensus Statement* (2015), and to present and discuss the outcomes of this harmonization initiative. It was recognized that physical literacy is a journey that continues across the lifespan; however, given the interest and scope of services of the participating organizations involved in the harmonization project, priority was given to physical literacy in the context of promoting healthy and holistic child development. This manuscript serves as a comprehensive and transparent account of the processes and outcomes of developing *Canada’s Physical Literacy Consensus Statement*, which aspired to provide a mechanism and impetus to clarify terminology, reduce confusion, increase alignment, and enhance synergy of effort among sector partners.

## Methods

**Fig. 1:**
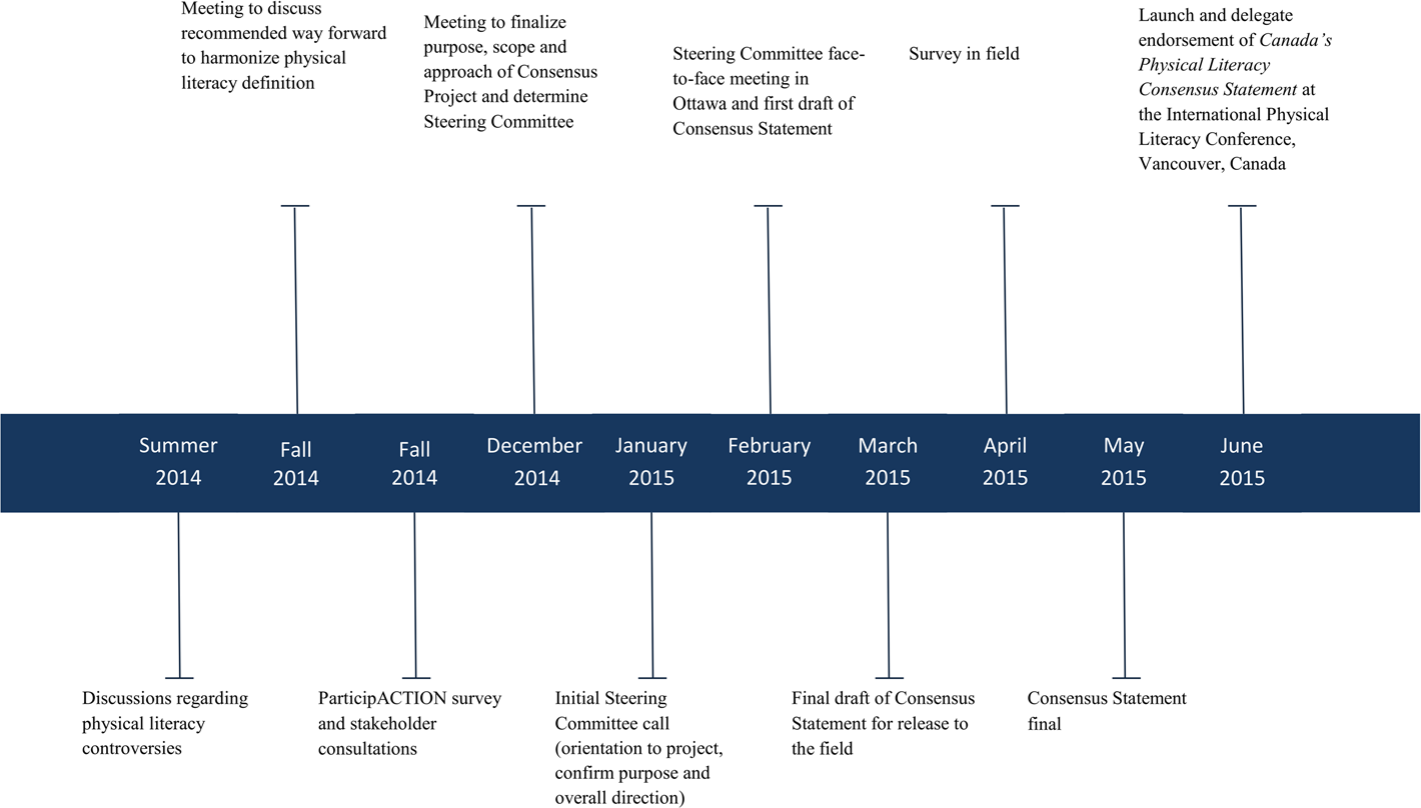
Major events in the development of *Canada’s Physical Literacy Consensus Statement*

A comprehensive, multi-stakeholder approach was used to pursue a common understanding and harmonization of physical literacy definitions and initiatives in Canada. The primary objective was to achieve a broadly supported definition of, and consensus statement for, physical literacy that would serve as a foundational document for the multiple sectors and stakeholders engaged in Canada’s physical literacy movement. This project occurred between 2014 and 2015 (as illustrated in Fig. 1) and included completing an environmental scan and stakeholder consultation, setting up a Steering Committee, developing a draft consensus statement, implementing a stakeholder survey to determine support for, and issues with, the draft consensus statement, and finally preparing and releasing *Canada’s Physical Literacy Consensus Statement*.

### Environmental scan

In addition to their commitment to develop a common definition of physical literacy, ParticipACTION also intended to enhance sector knowledge and understanding of existing initiatives (programs and resources) and identify the needs of the physical activity, sport, and recreation sectors related to physical literacy. To that end, they conducted an online survey (the Environmental Scan Survey) and a series of in-person consultations in the Fall of 2014 in order to:

1. gain insight into key messages, definitions of physical literacy, and related issues requiring clarity;
2. identify the types of programs/events/resources being developed; and
3. determine the needs of organizations to further their work in the area of physical literacy, including the need for a common definition [42].

Participants were recruited to participate in the online survey through:

- a promotional postcard that was disseminated via delegate bags and display booths at Physical Literacy Summits taking place just prior to or during the survey period;
- emails from ParticipACTION to their extensive partner network to distribute the survey to each of their partners’ networks;
- emails from ParticipACTION to Teen Challenge (www.participaction.com/en-ca/programs/participaction-teen-challenge) coordinators to distribute to their networks;
- information in the monthly ParticipACTION newsletter; and
- an email from S4L to their networks [42].

Participants were given 2.5 weeks to complete the survey. A copy of the Environmental Scan Survey is provided in Additional file 1.

Six in-person consultations were conducted in Alberta (2), Ontario, Manitoba, New Brunswick, and the Northwest Territories. Participants were invited based on recommendations from local and provincial/territorial organizations or were attendees at a related Physical Literacy Summit. Consultations occurring at conferences had a designated time allocation, and an open invitation to delegates was issued. Five of the consultations were led by ParticipACTION staff with the assistance of S4L consultants and/or the local or provincial organizer. One (Alberta) was led by a provincial partner organization (Ever Active Schools). Feedback was captured on flip charts and/or electronically. Three of the conversations were audiotaped [42].

### Steering committee formation and role

Concurrent to the survey and consultations, ParticipACTION met with researchers and a representative from S4L. It was agreed that a Steering Committee was essential to guide and inform the formulation of a common definition and Consensus Statement. The organizations and/or sectors well-known for their leadership in physical literacy were invited to participate, and accepted. The Steering Committee comprised representatives from HALO (research sector), S4L (sport sector), PHE Canada (education sector), the Canadian Parks and Recreation Association (CPRA) (recreation sector), OSPAPPH (public health sector), and the IPLA. It should be noted the two IPLA representatives contributed in-depth expertise in physical literacy with the intent to achieve a Canadian consensus statement with global input, rather than influencing/creating a global consensus statement. The Committee was chaired by ParticipACTION.

The Steering Committee met for the first time in January 2015 to learn the background of the initiative and to discuss the role of the Committee and its members. Key roles of Committee members included participating in Steering Committee meetings; developing the Consensus Statement and related document; participating in the development, implementation, and analysis of the Stakeholder Survey; planning the activation session at the International Physical Literacy Conference in June 2015; and disseminating the Consensus Statement throughout their respective networks. In February 2015, the Steering Committee conducted a half-day, face-to-face meeting in Ottawa, Canada, with the two IPLA members joining via conference call. All other meetings and communication were completed by email and conference calls.

Given the complex history of physical literacy development in Canada, the high expectations related to this process, and the skepticism of its success, building an influential Steering Committee to guide and inform the common definition / Consensus Statement development process was a pivotal part of the process. Membership was kept relatively small to facilitate a timely and efficient process, yet represented all of the key sectors currently engaged in physical literacy work. A list of Steering Committee members is provided in Table 1.

**Table 1:** List of Steering Committee members and affiliations

Steering Committee member	Affiliation
Brett Bartlett	ParticipACTION
Christa Costas-Bradstreet	ParticipACTION
Diana Dampier	ParticipACTION
Chantal Lalonde	Eastern Ontario Health Unit/Ontario Society of Physical Activity Promoters in Public Health
Reg Leidl	Physical and Health Education Canada
Brian Lewis	Physical and Health Education Canada
Melanie McKee	International Physical Literacy Association
Shelley Shea	Canadian Parks and Recreation Association
Liz Taplin	International Physical Literacy Association
Mark S. Tremblay	Healthy Active Living and Obesity Research Group, Children’s Hospital of Eastern Ontario Research Institute
Richard Way	Sport for Life Society

### Consensus statement development process

The proposed Consensus Statement development process was envisioned as involving the creation of a series of five to seven papers that would be prepared by leading physical literacy experts. The papers were planned to focus on historical background, constructs, controversies, and the different aspects of physical literacy, to inform delegates who would be invited to attend a Consensus Workshop to develop the final Consensus Statement.

Having agreed upon the topics for the papers, a conversation ensued that was premised on the proposal that the IPLA definition of physical literacy could provide the foundation of the Consensus Statement. Developed through an analysis of the evidence and a consensus process, the IPLA definition read as follows: “Physical literacy can be described as the motivation, confidence, physical competence, knowledge and understanding to value and take responsibility for engagement in physical activities for life.” (https://www.physical-literacy.org.uk/) It was unanimously decided that the project would take a new approach to build consensus on a definition of physical literacy in Canada. In preference to the proposed series of papers and a Consensus Workshop informing the development of a consensus statement, the Steering Committee decided to put forward the IPLA’s definition and write an accompanying consensus statement. This document would then be distributed for broad sector feedback in the form of a survey. That process would be followed by a Canadian endorsement of the definition and Consensus Statement at the International Physical Literacy Conference, taking place in June 2015 in Vancouver, Canada. Also proposed were activation sessions with practitioners from various sectors to determine the types of tools and resources that would be useful in communicating and understanding physical literacy within each sector.

The international research committee members representing the IPLA, working with the team from HALO, prepared a first draft of the Consensus Statement. Several rounds of input from the Steering Committee, through email and teleconferences, were solicited and subsequently refined the document. The draft Consensus Statement, titled *Canada’s Physical Literacy Consensus Statement*, presented a definition of physical literacy, a description of the four elements of the definition, and five overall physical literacy principles.

### Stakeholder survey

Following the adoption of the definition and the development of the Consensus Statement, and its translation to French, a stakeholder survey (developed by the research team and signed off by the Steering Committee) was used to collect input from stakeholders. Respondents were asked to provide feedback on the clarity of the Consensus Statement, as well as their level of agreement, perceived importance, and support for the Consensus Statement. Two additional questions appeared in the French survey, requesting feedback on the translation of the term “physical literacy”. A final question was included in the French and English surveys that allowed respondents to self-identify and choose to “endorse” the Consensus Statement. The stakeholder survey is provided in Additional file 2. The stakeholder survey was disseminated by Steering Committee members to their networks across Canada, reaching many stakeholders in the sport, recreation, physical activity, education, and public health/health promotion sectors. The survey was in the field for a period of 2.5 weeks (April 9–24, 2015). Further information was posted on the respective organization websites of Steering Committee members.

### Finalization, launch and Vancouver declaration

The survey results were provided to Steering Committee members, in both raw data and summary formats, for the purpose of making modifications to the Consensus Statement. The Steering Committee then met to review the results, and to make decisions and recommendations about what changes should be made and how they should be incorporated. ParticipACTION staff coordinated finalization of the Consensus Statement document with ongoing direction and involvement of Steering Committee members. The Consensus Statement document was finalized, translated into French, designed, and printed for paper and electronic distribution.

To further promote the Consensus Statement, the S4L team led the development of the “Vancouver Declaration”. This featured the common definition of physical literacy, all aspects of the Consensus Statement (four essential elements and five core principles), evidence-based facts supporting the need for attention on physical literacy, and a final declaration. This document was also translated into French, designed, and printed for electronic and paper distribution. The Vancouver Declaration was distributed to sector partners with a request to visit the ParticipACTION website (https://www.participaction.com) to or to endorse it at the/or to endorse it at the International Physical Literacy Conference for those attending. The Declaration was also printed on a large banner to be displayed and signed by delegates during the conference.

The official launch of *Canada’s Physical Literacy Consensus Statement* took place in Vancouver, Canada, at the 2015 International Physical Literacy Conference. Launch activities included an introduction to the Consensus Statement process during the opening ceremonies, a specific conference session dedicated to gathering feedback and input from delegates about additional communications materials, the arrangement of an activation area staffed by Steering Committee members in attendance at the conference, and a celebration at the closing ceremonies.

### Post-launch activities

The Consensus Statement was launched 2 years ago, allowing time for reflection and assessment of its impact at an organizational level. Organizations involved on the Steering Committee provided evidence that they have embraced, promoted, disseminated, and implemented the Consensus Statement into their work as a testimonial of the impact that the Consensus Statement has had on their organization (see Discussion).

## Results

### Environmental scan

The results of the Environmental Scan Survey and related consultations were summarized for ParticipACTION by the Propel Centre for Population Health Impact at the University of Waterloo [42]. Sixty-four respondents completed at least one of the consultation questions (72 completed the demographic section). The majority of respondents were between the ages of 25 and 55 years (85%), were female (71%), and had a Bachelor’s or Master’s degree (71%). Respondents represented all Canadian provinces/territories except Manitoba, New Brunswick, Nunavut, and Prince Edward Island. Respondents represented diverse sectors including sport (30%), public health/health promotion (27%), recreation (23%), education (23%), government (9%), youth serving agencies (5%), medical/allied healthcare professionals (3%), and health charity/consultant/social services (2%). Of those who indicated they were in the “other” category (17%), 6% were from the research sector.

The Environmental Scan Survey provided respondents with five definitions of physical literacy that could be selected based on their understanding of the term. Table 2 represents the findings from the survey. Respondents who chose the “other” category noted that various aspects of the different definitions informed their understanding of physical literacy. One respondent felt there were limitations with all of the definitions, particularly due to the fact that the definitions specifically referred to children rather than acknowledging that physical literacy is a lifelong pursuit.

**Table 2:** Environmental Scan Survey support for different definitions of physical literacy. (From Patton and Yessis, 2015 with permission)

Definition	Number (%) *n* = 64
Individuals who are physically literate move with competence and confidence in a wide variety of physical activities in multiple environments that benefit the healthy development of the whole person. Physically literate individuals consistently develop the motivation and ability to understand, communicate, apply, and analyze different forms of movement. They are able to demonstrate a variety of movements confidently, competently, creatively, and strategically across a wide range of health-related physical activities. These skills enable individuals to make healthy, active choices that are both beneficial to and respectful of their whole self, others, and their environment.	21 (32.8%)
Physical literacy is merely about developing the fundamental movement skills that all children need, such as running, hopping, throwing, catching and jumping. These movement skills in turn give kids the confidence to participate in different physical activities, sports, and games. Physical literacy is the mastering of fundamental movement skills and fundamental sport skills that permit a child to read their environment and make appropriate decisions, allowing them to move confidently and with control in a wide range of physical activity situations. It supports long-term participation and performance to the best of one’s ability.	12 (18.8%)
Physical literacy is the foundation of characteristics, attributes, behaviours, skills, awareness, knowledge, and understanding related to healthy active living and the promotion of physical recreation opportunities and positive health choices. Physically literate children learn from experiences in multiple domains (e.g., sport, physical education, play), multiple contexts (e.g., land, water, air, ice) and from multiple sources (e.g., coach, teacher, parent, peers).	11 (17.2%)
Physical literacy is the mastering of fundamental movement skills and fundamental sport skills that permit a child to read their environment and make appropriate decisions, allowing them to move confidently and with control in a wide range of physical activity situations. It supports long-term participation and performance to the best of one’s ability. Physical literacy is the cornerstone of both participation and excellence in physical activity and sport. Ideally, physical literacy is developed prior to the adolescent growth spurt. It has been adopted as the foundation of the Sport for Life concept in Canada. Children should learn fundamental movement skills and fundamental sport skills in each of the four basic environments: on the ground (as the basis for most games, sports, dance and physical activities); in the water (as the basis for all aquatic activities); on snow and ice (as the basis for all winter sliding activities); in the air – basis for gymnastics, diving and other aerial activities.	10 (15.6%)
Physical literacy can be described as the ability and motivation to capitalize on our movement potential to make a significant contribution to the quality of life. As humans we all exhibit this potential; however, its specific expression will be particular to the culture in which we live and the movement capacities with which we are endowed. An individual who is physically literate moves with poise, economy, and confidence in a wide variety of physically challenging situations. The individual is perceptive in ‘reading’ all aspects of the physical environment, anticipating movement needs or possibilities and responding appropriately to these, with intelligence and imagination. A physically literate individual has a well-established sense of self as embodied in the world. This, together with an articulate interaction with the environment, engenders positive self-esteem and self-confidence. Sensitivity to and awareness of our embodied capacities leads to fluent self-expression through non-verbal communication and to perceptive and empathetic interaction with others. In addition, the individual has the ability to identify and articulate the essential qualities that influence the effectiveness of his/her own movement performance, and has an understanding of the principles of embodied health, with respect to basic aspects such as exercise, sleep and nutrition.	7 (10.9%)
Other	3 (4.7%)

When asked about the issues and challenges in advancing physical literacy in their sector, respondents indicated a number of barriers. A total of 21 respondents indicated that *understanding of physical literacy* was an issue, with specific responses relating to the limited understanding of physical literacy by parents or the general public, and the lack of a common definition. *Value given to physical literacy or willingness to change* was cited by 11 respondents (17%). This theme was characterized by a lack of value given to physical literacy and, therefore, a lack of willingness or buy-in to change current programming/initiatives. *Cross-sector collaboration* was seen as a challenge by five respondents (8%), with a lack of coordination, duplication, and an inability to connect all the stakeholders through an effective delivery model given as examples. In fact, a large majority of survey respondents agreed (88% somewhat or strongly agreed, *n* = 56) that having a common definition of physical literacy would help them in their day-to-day work. Five respondents (8%) maintained that physical literacy programs were not accessible to people because of financial, transportation, or language barriers.

Survey respondents reported the physical literacy initiatives they were involved in developing/delivering/promoting, and which resources/tools they were using. Respondents were involved in delivering programs and integrating physical literacy into existing programs and practices, providing and supporting leadership training, assessment, developing partnerships, creating resources, research and evaluation, and advocacy. There was a very wide range of tools and resources in use (> 30 examples given); these tools and resources originated from the sport, education, recreation, physical activity, health, research, not-for-profit, and child development sectors, both within Canada and internationally. Similar diversity was observed with respect to physical literacy assessment methods: of the 41% who indicated they used an assessment method, 47% used the PLAY tools (S4L, www.physicalliteracy.ca/play-tools/), 42% used the Canadian Assessment of Physical Literacy (HALO, https://www.capl-eclp.ca), 21% used PHE Canada’s Passport for Life (www.phecanada.ca/resources/passport-for-life), and 21% indicated they used an “Other” tool. The majority of respondents (66%) somewhat or strongly agreed that further research about assessment methods was needed.

Respondents identified a number of gaps related to physical literacy resources/tools/information, including:

- *Information about physical literacy* (9 respondents, 14%). Respondents felt that physical literacy information could be improved, including its importance to specific sectors and “how to implement physical literacy” in various settings. They suggested a hub of information about best practices would be beneficial, and called for more information about the affective aspect, such as the motivation component of physical literacy. Five respondents (7%) suggested that additional research and evaluation were needed to assess whether current implementation models result in lifelong participation, and how assessment tools help to predict active healthy behaviour.
- *Collaboration and coordination in the physical literacy field* (9 respondents, 14%). They suggested that collaboration and coordination in the sector could be improved by building a community; using a bottom-up approach; sharing resources and information among sectors; working together; and developing a common implementation ideology.
- It was also noted by 8 respondents (12%) that both the general public and practitioners could benefit from education regarding physical literacy and the importance of a multi-sport approach.
- Resources were emphasized as gaps by 7 respondents (10%), who said that resources could be made more relevant and simple for those working at the grass-roots level. They reported that current resources are expensive and cumbersome to use; resources should be easily applied to a day-to-day practice; lesson plans would be helpful; and more resources are needed in French.
- Finally, a noted gap was the need for a universal assessment tool for physical literacy that would be targeted to all age groups (6 respondents, 9%).

### Stakeholder consultations

A total of six in-person consultations were conducted. Sectors represented included education, health, government, sport, recreation, and physical activity. There were between 15 and 30 participants in each consultation.

Participants were asked if/how having a common definition and/or understanding of physical literacy would help them in their day-to-day work, and whether or not having common key messages would be helpful in the communication of their physical literacy–based programs and initiatives. The responses indicated strong support for having common key messages and a common understanding of physical literacy, somewhat supporting the need for a common definition. Participants felt a common definition might be of more importance to researchers versus practitioners, but recognized its value in applying for funding and conducting assessments. Table 3 outlines the reasons for developing a common definition, understanding and/or key messages. Table 4 provides a summary of recommendations for developing key messages.

**Table 3:** Reasons to develop a common definition, understanding and/or key messages from stakeholder consultations

Reason	Examples from the discussions
Clarity of communication / understanding	There is a need for a common language so that everyone knows what we are talking about Right now we may think that we are talking about the same thing, but we may not be People get mixed messages from different definitions
Brand recognition / Generate buy-In	We can all speak the same language to decision makers and those who are not as familiar with the term and generate brand recognition A common understanding would help to generate buy-in with everyone we work with
Rationale for partnerships	A common definition will help to connect organizations, give reasons to engage and network
Support frontline staff	This will help with the interpretations at the front line
Consistency across sectors	Need consistent messaging across sectors
Give legitimacy to the sector	Give legitimacy for the sector
Enhance strategic planning	Service providers need a common definition to put into strategic plans and missions. This will then improve opportunities to collaborate
Consistency in assessment	There are inconsistencies in assessments of individual children Need a common way of measuring so we can all talk about the same thing A common definition can inform measurement tools
Common outcomes	No matter where you are in the country, province or city, you are getting the same outcomes

**Table 4:** Recommendations for the development of messages from stakeholder consultations

Factor to be considered	Examples from the discussions
Target populations	Definition / message should correspond with the audience Messages for a variety of audiences would be helpful Language background, age, sector, gender, region, parents, children, funders, culture, social inequities, health equity should all be considered One definition for within the sector, one for the general public Have a set definition with interpretations for various populations
Language	Plain language Literacy is a tough word for some people to understand The term literacy helps to bridge the construct across sectors
Sector-specific messaging / consistency across sectors	The “idea” needs to be consistent Consistency in branding of physical literacy, but message may change based on sector Success may look different in different sectors Each sector needs to have a key message Make the link to benefits / outcomes for specific sectors
General and specific messaging	A common definition for a global campaign with tools to talk to specific groups of people
Evidence-based	Whatever it is, it needs to be evidence-based
“Feel” of messages	Create urgency The message needs to be powerful Clearly share “this is what physical literacy is and how you get there” Use a strength-based approach with message Use physical literacy as a buzz term
Address embedded beliefs	Address the notion that “just get outside” is physical literacy
Format for messages	Images would be helpful Short bullet list of 5–6 key messages that can be picked from with a few key, foundational ones Develop “elevator talk” messages Short, sweet bits of information at a time
Process for developing messages	Messages should be developed from the top down
Message delivery	Would be great to have professional athletes involved in delivering messages
Message ideas	Physical literacy is about competence and confidence Physical literacy enhances health Physical literacy builds community Active start / active for life Physical literacy is lifelong / there is no endpoint Skills you can see / can you move properly? Physical literacy is more than just physical activity / fitness Physical literacy is accessible to all – there are lots of options beyond organized sport or that don’t cost money Unstructured and structured Physical literacy is the new social currency – leads to a willingness to try new things Reaching full potential / being able to participate fully in life

Those who did not feel a common definition was required reasoned that a common definition had either already been developed or was currently being developed, or that it would not be helpful. There were also concerns that developing a common definition could be fraught with challenges, including getting people together to do the work, the commitment to existing systems and definitions, and the risk of confusion from changing current definitions. It was also felt that the public’s and parents’ understanding of physical literacy would not be informed or changed by a new definition and all it entails. Other participants suggested an audit be done of what each organization was doing currently, while some felt that advancing a common definition would not matter because the organizations that employed differing definitions were communicating only with people who were working within their respective fields.

Similar to the Environmental Scan Survey, stakeholder consultation participants were asked to name the organizations they believed should be engaged in building a consensus around physical literacy terminology and a common definition. Responses appear in Table 5. Irrespective of the organizations involved, participants believed it was not in the best interest of the sector to reinvent the wheel or create something new. Other comments included building on the work of Active Canada 20/20 [43], the importance of having the federal government engaged in (but not leading) the work, the importance of having representatives from the provinces and territories engaged in any work happening at the national level, and the importance of having those who work in the physical literacy field take a leadership role.

**Table 5:** Key organizations that should be involved in building consensus around physical literacy terminology, a common definition and conceptual model from stakeholder consultations

Key organization / sector	Examples
Sport sector	Canadian Sport for Life; national, provincial, and municipal sport organizations; Sport Canada; Sport North; Sport Matters; provincial sport and recreation committees
Education sector	Ministries of Education; school boards and trustees; teachers’ associations; PHE Canada; Ever Active Schools
Government	Ministries of Education; municipal councils; school boards and trustees; school jurisdictions; teachers’ associations; government initiatives (such as Healthy U Alberta); provincial / territorial governments and relevant ministers; federal government
Active living sector	Be Fit for Life; ParticipACTION
Health sector	Healthcare providers; public health; doctors and physicians; health authorities (e.g., Alberta Health Services); epidemiology
Recreation sector	Provincial parks and recreation associations; outdoor groups and councils; recreation personnel associations; Canadian Parks and Recreation Association; provincial sport and recreation committees
Child care / early childhood sector	Early childhood educators
Universities / researchers	Students; professors; researchers; Alberta Centre for Injury Control Research (ACICR); Canadian Council of University Physical Education and Kinesiology Administrators; APPLE (Alberta Project Promoting Active Living and Healthy Eating) Schools (Alberta)
Non-governmental organizations	YMCA (Young Men’s Christian Association); Boys and Girls Club; non-profits involved in programming
Unions	Local unions
National organizations	National organizations (it was felt that the work should happen at this level); should be top-down from national organizations
Business / private sector	Publishers; Royal Bank of Canada
Parent groups	Alberta School Councils Association; Canadian Parents Association
Collaborative committees / community groups	Play groups; non-sport-related community groups
First Nations	Tribal councils
New Canadians	New Canadians
People with disabilities	People with disabilities

The stakeholder consultations were also designed to gather information about the work already taking place in each sector. Representatives were asked to share information about the programs, initiatives, tools, and resources they were working on or knew about. The findings reinforced the Environmental Scan Survey results, indicating that many and wide-ranging resources were being developed, used, and disseminated. These included projects, programs, training and learning opportunities, program adaptations, marketing and research, and evaluation initiatives in a variety of sectors and through new and existing partnerships. Post-secondary training (e.g., Mount Royal University Bachelor of Health and Physical Education with a Physical Literacy major), dedicated government staff positions, and funding for physical literacy initiatives were cited.

Assessment methods for evaluating physical literacy programs and initiatives that were mentioned in the stakeholder consultation meetings included S4L PLAY tools, PHE Canada – Passport for Life, CAPL, NCCP Fundamental Movement Skills (training program), Mount Royal University Physical Literacy Observation Tool (assesses at the level of a daycare, not individual children), Quest 2 Tool (High Five), gross motor skill assessment tools, and parent feedback, as well as registration, participation, and retention rates. Some of the points raised in the discussion of assessment instruments included: assessments needed to be simpler for younger populations; there was a need for clarity regarding which tool to use; each tool served a different purpose, assessed at different levels and provided different methods of feedback; users needed to know why they were doing the assessment and what the goal of the assessment was; assessment tools needed to be varied as the most appropriate tool depended on what was being taught and who the target population was; assessment should be standardized; and it was important to use the same tool for pre- and post-assessment. Suggestions for future research included: the long-term impact of physical literacy programming; developing tools for a younger-aged population; addressing physical literacy in adults; development of simple tools; clarifying what it means to be physically literate; more study on the emotional side of physical literacy; causal rather than correlational research; determining what the measure of success was for various population groups; community-level assessments to show how the community was doing in terms of physical literacy; use of physical literacy evidence to inform practice and policy; and evaluating how assessment data are being used.

Participants in the consultation sessions also discussed challenges to advancing physical literacy across sectors. The most notable challenges identified were the lack of awareness of the construct of physical literacy (both among the general public and within specific stakeholder groups including parents, politicians, and service providers); communicating the message about physical literacy using a branding strategy targeted specifically to the audience of interest; lack of coordination across sectors invested in physical literacy; competing priorities; and lack of best practices available for organizations to take action. Table 6 summarizes the sector-specific challenges that were shared through the stakeholder consultation meetings.

**Table 6:** Sector-specific challenges to advancing physical literacy identified during stakeholder consultations

Sector	Challenges
Education	Physical education teachers have no consultants/advisors Funding is limited for teacher release time Lack of in-services for physical literacy curriculum and lack of course profiles There are no accountability measures in place for physical education and physical literacy There is no “culture of physical activity” and decreased understanding of the benefits of physical activity Physical education is marginalized in comparison to numeracy and literacy; athletics gets the lion’s share of funding Teachers already have a full curriculum and full schedule
Municipal	Recreation staff lack awareness of physical literacy Funding and resources to improve understanding of physical literacy are limited Lack of champions for physical literacy
Sport	Provincial sport organizations don’t incorporate physical literacy into their strategic planning and don’t provide direction to counterparts The volunteer sport sector (such as parent coaches) is completely under-resourced
Political	Interested politicians sometimes feel like a lone voice for physical literacy

The specific needs articulated by respondents mirrored the challenges they reported facing in advancing physical literacy work, and were similar to the needs gathered through the Environmental Scan Survey. The major issues are listed in Table 7.

**Table 7:** Specific needs to advance physical literacy work identified in stakeholder consultations

Need	Examples
Resources / tools	Funding A built environment that supports physical literacy Time Tools for those working at the grassroots level
Communication / sharing / coordination	Sharing what is happening in the field and opportunities for partnership Success stories A framework of who is doing what for each population and at each stage Cross-sector collaboration to share messages with key decision makers Cross-sector integration that supports physical literacy from birth Conversations, sharing, and listening
Understanding / awareness of physical literacy	Education of the general population regarding physical literacy Community knowledge, understanding, and appreciation for the importance of physical literacy
Marketing / messaging	Advertisements A campaign that links physical health to learning ability A hub for messages and language Overarching messaging that includes all populations Translation of physical literacy to illustrate the importance of the construct to other sectors A common, understandable definition
Culture shift / physical literacy being valued	Relevant staff being given the authority and time to address physical literacy More advocates and champions A way to address systemic beliefs and practices regarding sport specialization and athletic development Buy-in from decision makers
Information / research	A system to understand how physical literacy supports literacy, numeracy, and mental health Tools, resources, and practices that satisfy multiple outcomes from across sectors Research regarding whether investments in physical literacy are paying off Research that supports a case for physical literacy Determination and communication of what the outcomes of physical literacy are Evidence to present to decision makers Translation of research for a lay audience
Assessment	Broader and clearer indicators for physical literacy assessment A self-assessment physical literacy app for hand-held devices
Programs	Programs from birth that are physical literacy based
Accountability	Mandatory time for physical education and physical literacy in schools A way to hold relevant stakeholders accountable for physical literacy
Leadership	Leadership from communities and municipalities A connection between school leadership and the community

Responses to questions about issues and challenges to implementing physical literacy programs and projects were similar to what had been discussed regarding broad challenges to advancing physical literacy and needs to advance physical literacy work, as presented above. The major themes included resources, programming challenges, understanding physical literacy, an emphasis on sport and athletics, definition/branding/marketing issues, and relative value compared to competing priorities.

Relationship-building was the most mentioned way of working together in the sector. In some cases, participants developed formal partnership agreements, while others worked with organizations that were interested in collaborating. Participants identified a number of partners from different sectors with whom they are working including non-government, government, health, and education organizations. Collaboration took place by developing joint use agreements; partnering on grant applications; forming community coalitions; having common agendas; advancing grassroots collaborations; attending or hosting multi-sector events; and jointly developing policy. Challenges of working with partners included the optics of working with a for-profit organization, particularly when a corporate sponsor was involved, and the need for government support of physical literacy. Developing further partnerships with non-traditional groups (such as cultural groups, dance groups, music groups, and corporate partners) was suggested.

### Stakeholder survey

During the 2.5 weeks the online stakeholder survey was open, 2243 respondents landed on the front page. Responses varied by question (1313–1374 responses for close-ended questions; 486 responses for open-ended questions), with lower completion rates on questions in the middle to end of the survey. Respondents were from every province and territory, with the majority living in Ontario (35%), Alberta (16%), British Columbia (14%), Manitoba (8%), and Québec (7%). International respondents (4%) were from other parts of North America (United States, St. Vincent and the Grenadines), South America (Brazil), Europe (United Kingdom, Ireland, Sweden, Italy, Portugal, Slovenia), Asia (Jordan, Qatar, Hong Kong), and Oceania (Australia, New Zealand). The majority of respondents represented the education (30%), sport (20%), recreation (11%), and physical activity/fitness (10%) sectors.

For all sections of the draft of *Canada’s Physical Literacy Consensus Statement* (purpose, definition, elements, principles), the proportion of respondents who strongly agreed or somewhat agreed that the sections were clearly stated ranged from 89 to 96%. The proportion who strongly agreed or somewhat agreed with the content of the message in these sections ranged from 87 to 96% (Table 8). Results were similar regardless of the geographic location of the respondent or their sector (see Additional file 3 for details).

**Table 8:** Stakeholder assessment of the Consensus Statement’s clarity and stakeholder level of agreement, by section (*n* [%])

Section	Section clearly stated				Agreement with section content			
	Total n	Strongly agree	Somewhat agree	Combined agreement	Total n	Strongly agree	Somewhat agree	Combined agreement
Purpose	1374	847 (61.6%)	444 (32.3%)	1291 (93.9%)	1369	947 (69.2%)	358 (26.2%)	1305 (95.4%)
Definition	1370	742 (54.2%)	480 (35.0%)	1222 (89.2%)	1366	708 (51.8%)	482 (35.3%)	1190 (87.1%)
Elements	1368	961 (70.2%)	336 (24.6%)	1297 (94.8%)	1365	927 (67.9%)	330 (24.2%)	1257 (92.1%)
Principles	1332	996 (74.8%)	280 (21.0%)	1276 (95.8%)	1333	1032 (77.4%)	247 (18.5%)	1279 (95.9%)

Four hundred and eighty-six participants provided comments to the open-ended questions on the survey. While the relative number of comments was low, there were some general consistencies. The common themes related to the comments are as follows:

- 16% (*n* = 79) of participants’ comments agreed with the Consensus Statement and proposed definition, praised the consensus process, or felt that this initiative was important and were supportive.
- 23% (*n* = 112) of participants’ comments indicated that the definition required modification, including suggested revisions such as: remove the word “responsibility” since it seldom applies to children (as parents typically dictate their child’s activities at a younger age); include wording relevant to children with disabilities; highlight accessibility issues; provide age-specific goals; provide examples of activities for different stages of development; include the environments in which physical activity occurs (e.g., water, ground, ice).
- 32% (*n* = 155) believed that the Consensus Statement was too complex, and that simplifying/rewording it would make it easier to understand for the entire population.
- 23% (*n* = 110) of participants provided miscellaneous comments.

The Steering Committee reviewed each of the themes overall, considered the individual comments, and decided how to address the issues raised. Table 9 summarizes the common themes, and describes how the Committee incorporated them into the Consensus Statement and supporting documents.

**Table 9:** Participant comments about the Consensus Statement, with Steering Committee decisions/actions

Themes from participant comments	Steering Committee decisions and actions
There were many comments related to the “readability” / literacy level / wordiness of the statement and the elements	Agreed that the Consensus Statement is meant for practitioners. Determined that the communication materials would take the Consensus Statement and bring it to life and that it would be presented in a different formats that will address the needs of specific sectors. The Consensus Statement would be tweaked to enhance its readability (vs literacy level). Miscellaneous comments would be addressed when the communications materials were produced. The Consensus Statement was sent to an experienced copy editor for comments/edits.
Many comments discussed the challenges related to implementation as opposed to the definition of physical literacy itself (socio-economic status, funds required, decision makers’ support, etc.)	These comments were to be considered in the development of the communications materials.
Many comments were concerned about the ‘personal responsibility’ aspect and the ‘value’ – the Consensus Statement seemed to many to be more directed to adults rather than kids	The reference to ‘personal responsibility’ needs discussion in the manuscript (this paper), as it relates to a person’s different stages of life (e.g., in the case of children, significant others share the responsibility).
There were some comments that the elements should be reordered, with physical being first	It was agreed to format the document in two ways and then determine the best way of presenting the information in a neutral way: (1) present as a list, but remove the letters in front of the words (suggesting an order) and (2) present in a four-column format. In the end it was decided to list in the order they are mentioned in the definition. Wording added to the introductory sentence that emphasizes that fact that all elements are of equal importance, but that the relative importance may vary throughout one’s life.
Many had a hard time with the “affective” element, particularly related to motivation. Further, many did not think we captured the “enjoyment” aspect	The concern re “motivation” was discussed and it was agreed that this is a seminal construct in the definition of physical literacy. The definition was changed to: “Motivation and confidence refers to an individual’s enthusiasm, enjoyment of and self-assurance to ...”.
There were comments asking that the Consensus Statement bring in the contribution of positive experiences and address overall development as a whole person / holistic approach	The comments related to positive experiences would be addressed in messaging and implementation materials and, therefore, would not be added into the Consensus Statement. With regard to the notion of the whole person – a sentence would be added to the core principles (as #5): “Physical Literacy contributes to the development of the whole person.” The manuscript (this paper) would discuss the whole person/holistic approach / physical literacy’s contribution to health and wellness.
Many comments addressed what currently appears in the Core Principles – need to explain the information more clearly	Left as is, with 2 changes: Add a 5th bullet Bullet 3 will now read: “Should be cultivated and enjoyed through a range of experiences in different environments and contexts”

### Finalization, launch and Vancouver declaration

**Fig. 2:**
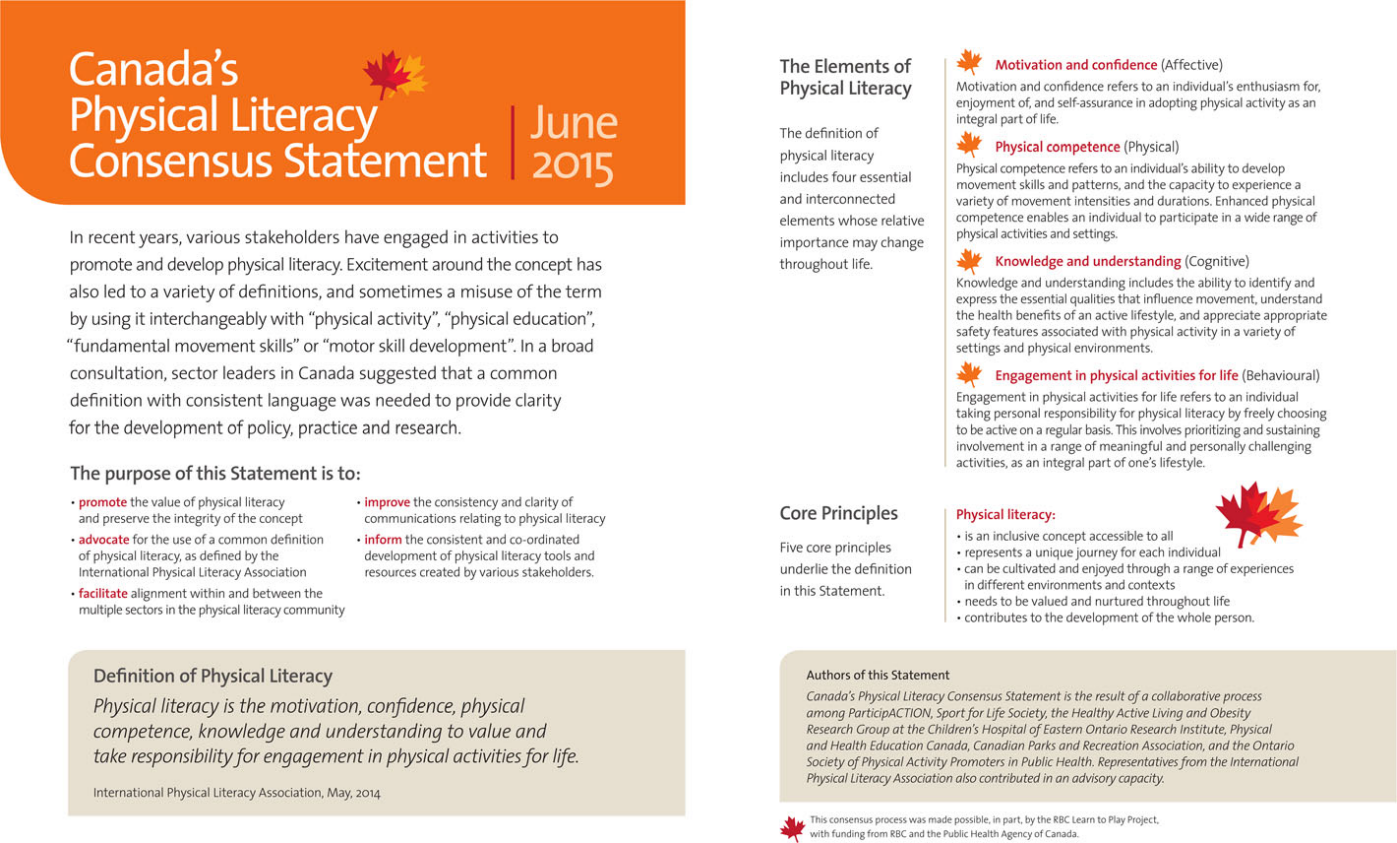
*Canada’s Physical Literacy Consensus Statement*

**Fig. 3:**
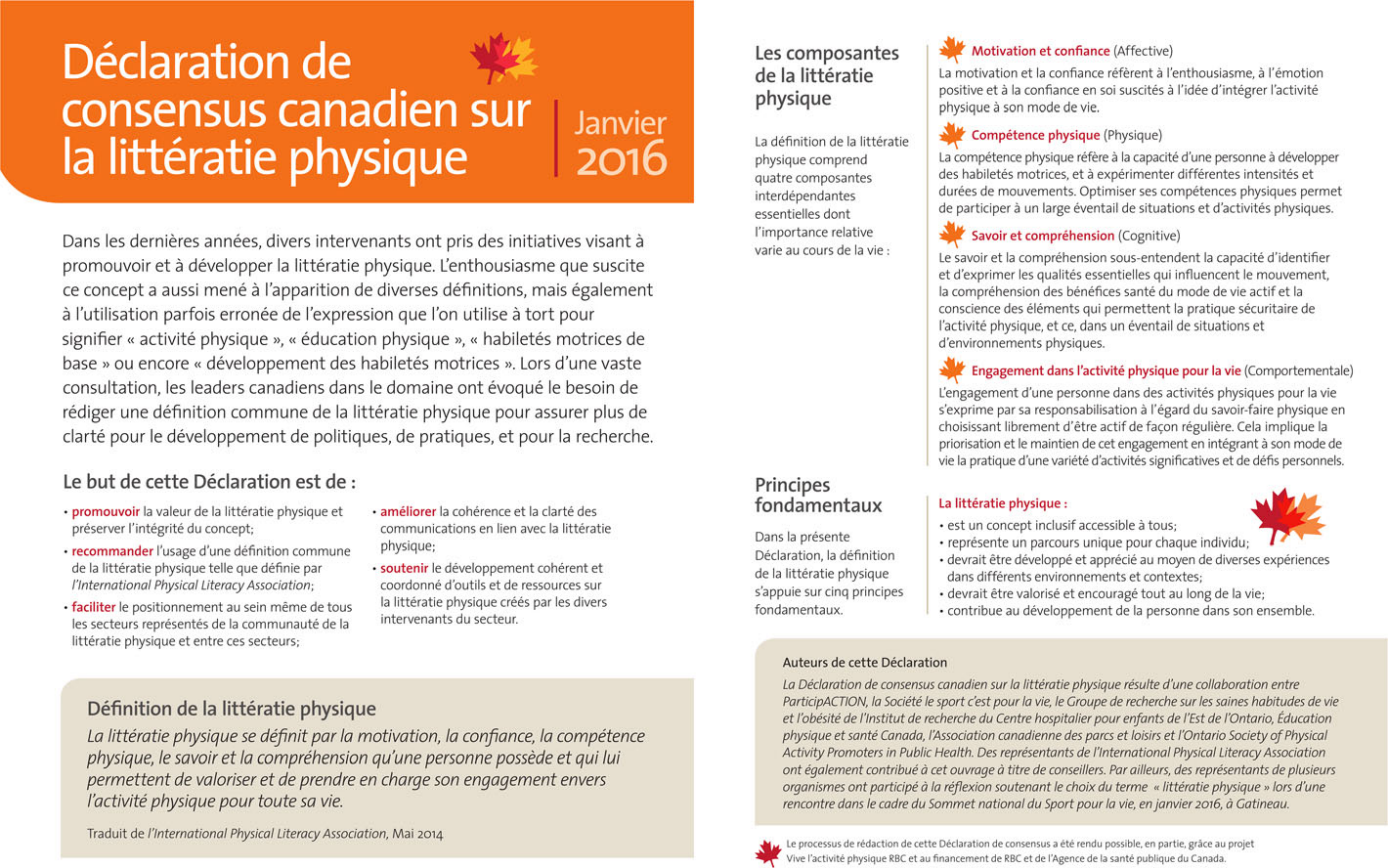
*Déclaration de consensus canadien sur la littératie physique* (French version of Canada’s Physical Literacy Consensus Statement)

The final product – *Canada’s Physical Literacy Consensus Statement* [44] – is provided in Fig. 2 (English) and Fig. 3 (French). At the conclusion of the process, all of the organizations represented on the Steering Committee endorsed and supported the Consensus Statement.

Survey respondents had been asked to indicate their interest in being supporters of the Consensus Statement once it was finalized, and 736/1313 (56.1%) indicated they would like to be contacted for final review so they could decide whether to be listed as a supporter. The Consensus Statement was sent out electronically and distributed via social media through the different networks of the Steering Committee members. As well, printed copies were provided to all International Physical Literacy Conference 2015 delegates and sent to subsequent conferences upon request. Copies of the Consensus Statement were distributed and incorporated into presentations by members (and related individuals/organizations).

The official launch of *Canada’s Physical Literacy Consensus Statement* took place in Vancouver, Canada, at the 2015 International Physical Literacy Conference. At the opening ceremonies, an introduction to the Consensus Statement (why it was produced, how, etc.) was presented; the President of ParticipACTION and the Chief Executive Officer of S4L signed the Vancouver Declaration banner; and delegates were encouraged to sign the banner during their time at the conference. Over the 3 days of the conference, delegates were invited to visit the Consensus Statement activation area (display) hosted by Steering Committee members; 400 delegates signed the banner, and 140 registered their endorsement on computers at the activation area. Throughout the 3 days, input from all delegates was sought regarding the types of resources, key messages, and other initiatives they would like to access to support their work related to physical literacy. They shared their comments on large poster boards at the activation area and at a consultation that was part of the conference program. The conference concluded with Dr. Margaret Whitehead, President of the IPLA, signing the Vancouver Declaration banner.

## Discussion

Physical literacy is made up of component parts that are not new, but as an over-arching construct it has inspired both great enthusiasm and debate since it began to garner attention through the teachings of Whitehead [29]. Physical literacy has evoked excitement and passion both in Canada and worldwide in the physical activity promotion movement. It is a construct that has influenced the work of a diverse number of sectors including physical activity, sport, recreation, education, and public health, and has served as a conduit to unite sectors. In Canada it has captured the imagination of leaders at the municipal (urban and rural), provincial, territorial, and national level. From the non-profit/non-government organization (NGO) sectors to the business community, people could envisage a role for themselves and their organizations in enhancing physical literacy levels for the purpose of increasing lifelong participation in physical activity and sport.

The emergence of the physical literacy movement prompted widespread and rapid growth in the areas of program and resource development. In an effort to define physical literacy in a way that reflected, supported, and promoted the mandate of each sector, a variety of definitions emerged that were relevant to specific sectors. While similarities existed, the different definitions led to confusion within and across sectors.

As evidenced by the findings from the two different surveys (Environmental Scan Survey and Stakeholder Survey) and the consultations, support for a common definition and common elements of the construct of physical literacy was identified as a foundation to enhance coordination and communication across the country in a way that would support practitioners and inform the general public, especially parents (e.g., so there is an understanding of what the term means when it is used by their child’s coach, physical education teacher, playground supervisor, etc.). The need for a common definition was identified across survey questions and consultations, and was reinforced in Steering Committee discussions. The issue of having a common definition that was relevant to all practitioners was raised in both the surveys and during the consultations. Therefore, there was an obligation to ensure that all practitioners could see how the definition of physical literacy, its elements, and its principles influence their work.

Working toward a more collaborative approach to enhancing physical literacy was a common theme and involved having access to more opportunities to communicate; to share information, resources and tools; to coordinate initiatives; and to learn from each other. Respondents also suggested the notion of a central hub, which could be a forum to share best practices, success stories, and training tools. Key to the success of such initiatives was a common understanding of the construct of physical literacy.

An issue that was both explicitly stated and subtly referenced in the surveys, consultations, and Steering Committee meetings was the issue of “in-fighting” or the notion of having different “camps” in Canada when it came to physical literacy resources, training, and evaluation. Ultimately, better alignment among the diverse sectors and organizations was the desired outcome, which many indicated would be facilitated by the Consensus Statement.

A great deal of information was collected about the needs and gaps that each sector sees as needing to be filled in order to enhance physical literacy levels in Canada. Survey participants, when asked what support they needed, requested resources that gave information on “how to implement physical literacy”. The environmental scan and stakeholder consultations demonstrated the need to raise awareness of the importance of physical literacy in general, and to tailor messages and provide information to specific sectors about how to support and enhance the development of physical literacy. It is the responsibility of sectoral leaders to take these requests and determine how to support their respective sectors. An encouraging start is having a common definition and understanding of physical literacy. It will now require a concerted effort, from all organizations and sectors involved, to begin to share the Consensus Statement with stakeholders and to ensure that the Statement is the foundation upon which their programs and resources are developed moving forward.

Since the release of the Consensus Statement, there has been evidence that sectors represented on the Steering Committee have embraced, promoted, disseminated, and implemented the Consensus Statement into their work. Below are a few examples.

- More than 200 organizations and individuals endorsed the Consensus Statement on ParticipACTION’s website.
- S4L and the Coaching Association of Canada facilitated the development of the National Physical Literacy Alliance (NPLA), consisting of more than 40 national/provincial organizations (from grass roots to podium) that are committed to ensuring all Canadians are healthy and active. This alliance seeks to ensure that all Canadians are competent, confident, and motivated to remain physically active for their entire lives. One of the key areas of work of the NPLA is communications, and a sub-committee was formed (led by ParticipACTION) to support the development of communication tools targeted to the broad physical activity sector to communicate the elements of the Consensus Statement in a consistent and coordinated way. RBC Learn to Play and PHAC supported the NPLA’s development, and ParticipACTION and the sub-committee members disseminated the tools through their networks, at conferences, and through their communication channels. The following link houses all the physical literacy resources including the Consensus Statement, an infographic, a key messages document based on the Consensus Statement, and a social media kit (https://www.participaction.com/en-ca/thought-leadership/physical-literacy).

While the process to create the Consensus Statement was comprehensive, thorough, and inclusive, there were some important limitations of the process. First, from inception it was understood by the Steering Committee that the focus was on children and youth versus adults. Although the Consensus Statement acknowledges that physical literacy is a lifelong experience, the Consensus Statement process was more focused on children and youth and their healthy development, consistent with articulated priorities in Canada [13–16]. Moving forward, the definition should be periodically revisited, and if necessary updated, to ensure it conveys physical literacy as a lifelong journey, and perhaps to determine if and what changes are required for adult and older adult audiences. Second, generalizations from the environmental scan and in-person consultations must be made with caution because of the potential for response bias.

Although all survey materials were delivered in both English and French, there was some concern from the Francophone population, particularly in Québec, that the process did not adequately engage them, particularly with respect to the definition of physical literacy in French. While feedback from respondents in Québec about the definition in French was received and incorporated, since the release of the Consensus Statement there has been considerable additional discussion regarding which terms were acceptable by Francophone leaders, primarily in Québec. At the 2016 S4L Summit, there was a workshop on the Consensus Statement and the French translation for the Francophone community, including 30 organizations from Québec and one from New Brunswick, where there was agreement in principle on the statement and its translation. However, the specific term (savoir-faire physique) used in the translation of the Consensus Statement ultimately was NOT accepted. After much dialogue the following was accepted “*La littératie physique est la motivation, la confiance, la compétence physique, le savoir et la compréhension qu’une personne possède et qui lui permettent de valoriser et de prendre en charge son engagement envers l’activité physique pour toute la vie*”*.* The Government of Québec has since included physical literacy in its provincial physical activity strategy, and a key, multi-organization committee focused on motor skill development has adopted the new definition and terms.

## Conclusions

In *Canada’s Physical Literacy Consensus Statement*, physical literacy is defined as the motivation, confidence, physical competence, knowledge and understanding to value and take responsibility for engagement in physical activities for life. Sector partners hope that the Consensus Statement will bring greater harmony, synergy, and consistency to physical literacy efforts both in Canada and internationally.

## Additional files

Additional file 1: Environmental Scan Survey. (DOCX 19 kb)

Additional file 2: Stakeholder Survey. (DOCX 137 kb)

Additional file 3: Detailed results from the Stakeholder Survey. (DOCX 50 kb)

## Abbreviations

CAPL: Canadian Assessment of Physical Literacy
CPRA: Canadian Parks and Recreation Association
HALO: Healthy Active Living and Obesity Research Group
IPLA: International Physical Literacy Association
MVPA: Moderate to vigorous physical activity
NCCP: National Coaching Certificate Program
NGO: Non-government organization
NPLA: National Physical Literacy Alliance
OSPAPPH: Ontario Society of Physical Activity Promoters in Public Health
PHAC: Public Health Agency of Canada
PHE Canada: Physical and Health Education Canada
PLAY: Physical Literacy Assessment for Youth
RBC: Royal Bank of Canada
S4L: Sport for Life Society
