# Supplementary material for: Canada’s Physical Literacy Consensus Statement: process and outcome
Source: BMC Public Health. 2018 Oct 2;18(Suppl 2):1034. doi: 10.1186/s12889-018-5903-x (PMC6167775; doi:10.1186/s12889-018-5903-x)
Supplement: Supplementary file 1 — Environmental Scan Survey. (DOCX 19 kb) [file 12889_2018_5903_MOESM1_ESM.docx]

1. What is your age?
   1. 18-24
   2. 25-34
   3. 35-44
   4. 45-54
   5. 55-64
   6. 65+
2. What is your gender?
   1. Male
   2. Female
3. What is the highest degree or level of school you have completed? If currently enrolled, highest degree received?
4. Some high school, no diploma
5. High school graduate, diploma or the equivalent
6. Trade, technical, or vocational training
7. College degree
8. Bachelor’s degree
9. Master’s degree
10. Doctorate degree
11. What province/territory are you from?
12. Alberta
13. British Columbia
14. Manitoba
15. New Brunswick
16. Newfoundland and Labrador
17. Northwest Territories
18. Nova Scotia
19. Nunavut
20. Ontario
21. Prince Edward Island
22. Quebec
23. Saskatchewan
24. Yukon Territory
25. What sector best describes your organization? (Check all that apply)
26. Youth Serving Agency
27. Education (Schools, school boards, post-secondary)
28. Recreation
29. Medical/Allied Health Care Professional
30. Social Services
31. Health Charity
32. Public Health and Health Promotion
33. Sport
34. Government
35. Consultant
36. Other (please specify)
37. Define physical literacy as you understand it:
38. Physical Literacy is the mastering of fundamental movement skills and fundamental sport skills that permit a child to read their environment and make appropriate decisions, allowing them to move confidently and with control in a wide range of physical activity situations. It supports long-term participation and performance to the best of one’s ability. Physical Literacy is the cornerstone of both participation and excellence in physical activity and sport. Ideally, physical literacy is developed prior to the adolescent growth spurt. It has been adopted as the foundation of the Sport for Life concept in Canada. Children should learn fundamental movement skills and fundamental sport skills in each of the four basic environments; On the ground (as the basis for most games, sports, dance and physical activities) In the water (as the basis for all aquatic activities); On snow and ice (as the basis for all winter sliding activities); In the air – basis for gymnastics, diving and other aerial activities.
39. Physical literacy is merely about developing the fundamental movement skills that all children need, such as running, hopping, throwing, catching and jumping. These movement skills in turn give kids the confidence to participate in different physical activities, sports, and games. Physical Literacy is the mastering of fundamental movement skills and fundamental sport skills that permit a child to read their environment and make appropriate decisions, allowing them to move confidently and with control in a wide range of physical activity situations. It supports long-term participation and performance to the best of one’s ability.
40. Individuals who are physically literate move with competence and confidence in a wide variety of physical activities in multiple environments that benefit the healthy development of the whole person. Physically literate individuals consistently develop the motivation and ability to understand, communicate, apply, and analyze different forms of movement. They are able to demonstrate a variety of movements confidently, competently, creatively and strategically across a wide range of health-related physical activities. These skills enable individuals to make healthy, active choices that are both beneficial to and respectful of their whole self, others, and their environment.
41. Physical literacy can be described as the ability and motivation to capitalize on our movement potential to make a significant contribution to the quality of life. As humans we all exhibit this potential, however its specific expression will be particular to the culture in which we live and the movement capacities with which we are endowed. An individual who is physically literate moves with poise, economy and confidence in a wide variety of physically challenging situations. The individual is perceptive in ‘reading’ all aspects of the physical environment, anticipating movement needs or possibilities and responding appropriately to these, with intelligence and imagination. A physically literate individual has a well-established sense of self as embodied in the world. This together with an articulate interaction with the environment engenders positive self-esteem and self-confidence. Sensitivity to and awareness of our embodied capacities leads to fluent self-expression through non-verbal communication and to perceptive and empathetic interaction with others. In addition, the individual has the ability to identify and articulate the essential qualities that influence the effectiveness of his/her own movement performance, and has an understanding of the principles of embodied health, with respect to basic aspects such as exercise, sleep and nutrition.
42. Physical literacy is the foundation of characteristics, attributes, behaviours, skills, awareness, knowledge and understanding related to healthy active living and the promotion of physical recreation opportunities and positive health choices. Physically literate children learn from experiences in multiple domains (e.g. sport, physical education, play), multiple contexts (e.g. land, water, air, ice) and from multiple sources (e.g. coach, teacher, parent, peers).
43. Other (please specify)
44. What are some of the issues/challenges to advancing physical literacy in your sector?
45. To what extent do you agree that having a common definition and/or understanding of the elements of physical literacy help you in your day-to-day work?
    1. Strongly agree
    2. Somewhat agree
    3. Somewhat disagree
    4. Strongly disagree

If disagree, please explain why

9. To what extent do you agree there is a role for ParticipACTION to help identify and communicate common terms and key elements related to physical literacy?

a. Strongly agree

b. Somewhat agree

c. Somewhat disagree

d. Strongly disagree

10. Which key organizations should be involved in helping to build consensus around physical literacy terminology, an operational definition and an overarching model for physical literacy? Consider NGO / government / sectors / levels.

11. Please describe the Physical Literacy initiatives are you involved in developing /delivering / promoting.

12. What resources / tools are you currently using? Please include hyperlinks to the resources if you are willing to share them.

14. Do you currently use a Physical Literacy assessment method? Yes____ No_____If yes, which one?

15. To what extent do agree further research about assessment methods is required?

1. Strongly agree
2. Somewhat agree
3. Somewhat disagree
4. Strongly disagree

16. What gaps (if any) exist related to physical literacy resources/tools/information?

17. What do you need, specifically, to help you do your work in the area of physical literacy?

18. With which sectors do you partner, with respect to your physical literacy initiatives? [physical activity, sport, recreation, education, public health, government partners, other (please specify)]

19. What support would you need (if any) to more effectively communicate with/work with/engage other sectors?

20. Would you be willing to share some of the work you are doing, including the partners / organizations with whom you work and the people that you serve / target with your work?

21. Please provide us with your contact information for a chance to win a FitBit.

1. Name
2. Email
