# Supplementary material for: Canada’s Physical Literacy Consensus Statement: process and outcome
Source: BMC Public Health. 2018 Oct 2;18(Suppl 2):1034. doi: 10.1186/s12889-018-5903-x (PMC6167775; doi:10.1186/s12889-018-5903-x)
Supplement: Supplementary file 2 — Stakeholder Survey. (DOCX 137 kb) [file 12889_2018_5903_MOESM2_ESM.docx]

Canada's Physical Literacy Consensus Statement

#

**Study title:** Opinion Survey for Canada’s Physical Literacy Consensus Statement

**Investigators:**

- Dr. Mark Tremblay, Healthy Active Living and Obesity Research Group, Children’s Hospital of Eastern Ontario (CHEO), email: [mtremblay@cheo.on.ca](mailto:mtremblay@cheo.on.ca)

As a practitioner or researcher whose work is in some way connected with physical literacy and/or the promotion of physical activity, sport and recreation, you are being invited to participate in a survey soliciting your opinion on a draft of Canada’s Physical Literacy Consensus Statement. 
 
Increasing interest in physical literacy among various stakeholders has led to many developments in advancing its promotion and support through a proliferation of initiatives. Excitement around the emergence of this new term has also led to a variety of definitions, and sometimes a misuse of the concept by using it interchangeably with terms such as ‘physical activity’, ‘physical education’, ‘fundamental movement skills’ or ‘motor skill development’. Consultation with sector leaders in Canada revealed that a common definition was needed and desired. A uniform definition with consistent language would provide clarity for the development of policy, practice and research.
 
The Consensus Statement has been prepared by a Steering Committee comprised of ten individuals from seven organizations with both a Canadian and international perspective*. These organizations, coming with various perspectives and definitions of physical literacy, convened to examine a process to develop a Consensus Statement.  What resulted was agreement to put forward the definition developed by the International Physical Literacy Association, which is based on both evidence and consultations with a variety of sectors.
 
This Consensus Statement presents a definition of Physical Literacy as well as a description of the elements of the definition.  As the final stage in the development of this Consensus Statement we are seeking to gain feedback about the clarity of the Consensus Statement, as well as level of agreement, perceived importance, and support for the Consensus Statement from a large number of practitioners in the fields represented by each of the contributing collaborators (e.g., sport, recreation, education, public health, health promotion, physical activity, etc.).
 
Acceptance of a common definition is important for the alignment of strategic efforts in policy, practice, and research aimed at promoting physical literacy.  Following this broad stakeholder consultation and subsequent revisions, the final Consensus Statement will be launched at the International Physical Literacy Conference in Vancouver, British Columbia, Canada in June, 2015. Further discussions will lead to the production and publication of a more detailed background document on the process and outcomes related to the production of the Consensus Statement.

Participation in this survey is voluntary. By accessing and completing this survey you are giving your implied/passive consent to participate in the survey. The survey does not collect information about your name or email address and responses will be presented in group format only. A summary of findings will be available on the ParticipACTION website (www.ParticipACTION.com) once the results have been published and will also be sent to participating organizations for distribution to members. If you have any questions about this study, please contact Dr. Mark Tremblay at 613-737-7600 ext 4114 or mtremblay@cheo.on.ca. The Children’s Hospital of Eastern Ontario (CHEO) Research Ethics Board (REB) has reviewed this protocol. The REB considers ethical aspects of all research studies involving human participants at the CHEO and its Research Institute. If you have any questions about your rights as a study participant, you may contact the CHEO REB Chairperson at 613-737-7600 ext 3624. Note: Canada’s Physical Literacy Consensus Statement is in draft form and not intended for general circulation.
 
Click on the link below for more information on the survey and instructions on how to get started.  Thanks for your time!
 
<http://fluidsurveys.com/s/physical-literacy/>
 
**We encourage you to circulate the survey link to your colleagues and among your networks. This stakeholder survey will be open from April 9 until April 24, 2015.**
 
*Canada’s Physical Literacy Consensus Statement is the result of a collaborative process between ParticipACTION, Sport for Life Society, the Healthy Active Living and Obesity Research Group at the Children’s Hospital of Eastern Ontario Research Institute, PHE Canada, Canadian Parks and Recreation Association, and the Ontario Society of Physical Activity Promoters in Public Health. Representatives from the International Physical Literacy Association also contributed in an advisory capacity.

# Canada's Physical Literacy Consensus Statement

With increasing interest in physical literacy among various stakeholders, there have been many developments in advancing its promotion through a proliferation of initiatives. Excitement around the emergence of this concept has also led to a variety of definitions, and sometimes a misuse of the term by using it interchangeably with ‘physical activity’, ‘physical education’, ‘fundamental movement skills’ or ‘motor skill development’. Consultation with sector leaders in Canada revealed that a common definition was needed and desired. A uniform definition with consistent language would provide clarity for the development of policy, practice and research. This Statement aims to: Promote the value of physical literacy and preserve the integrity of the concept; Advocate for the use of a common definition of physical literacy as defined by the International Physical Literacy Association; Facilitate strategic alignment within and between the multiple sectors represented in the physical literacy community; Provide guidance to improve the consistency and clarity of communications relating to physical literacy; and Inform the consistent and co-ordinated development of physical literacy tools/resources created by various stakeholders.

# 1. Is the purpose of the consensus statement clearly stated?

|  | Strongly Agree |
| --- | --- |
|  | Somewhat Agree |
|  | Neither Agree Nor Disagree |
|  | Somewhat Disagree |
|  | Strongly Disagree |

# 2. Do you agree with the purpose of the consensus statement?

|  | Strongly Agree |
| --- | --- |
|  | Somewhat Agree |
|  | Neither Agree Nor Disagree |
|  | Somewhat Disagree |
|  | Strongly Disagree |

# Definition of Physical Literacy

Physical literacy is the motivation, confidence, physical competence, knowledge and understanding to value and take responsibility for engagement in physical activities for life.- International Physical Literacy Association

# 3. Is the definition clearly stated?

|  | Strongly Agree |
| --- | --- |
|  | Somewhat Agree |
|  | Neither Agree Nor Disagree |
|  | Somewhat Disagree |
|  | Strongly Disagree |

# 4. Do you agree with the definition?

|  | Strongly Agree |
| --- | --- |
|  | Somewhat Agree |
|  | Neither Agree Nor Disagree |
|  | Somewhat Disagree |
|  | Strongly Disagree |

# The Elements of Physical Literacy

The definition of physical literacy comprises four elements that intersect and overlap: a. Affective - “motivation and confidence”. Motivation and confidence refers to an individual’s enthusiasm, excitement, and self-assurance to adopt physical activity as an integral part of life which is the fundamental aspiration of practitioners working to promote physical literacy. b. Cognitive - “knowledge and understanding”. Knowledge and understanding includes the ability to identify and articulate the essential qualities that influence movement performance, understanding the health benefits of an active lifestyle and appreciating appropriate safety features associated with physical activity in a variety of settings and different physical environments. c. Physical - “physical competence”. Physical competence refers to an individual’s ability to develop movement skills and patterns, and establishing the capacity to experience a variety of movement intensities and durations.  Enhancement of physical competence will enable the individual to participate in a wide range of physical activities and settings. d. Behavioural - “engagement in physical activities for life”. Engagement in physical activities for life pertains to an individual taking personal responsibility for physical literacy, resulting in them freely choosing to be habitually active. They will prioritise and sustain their involvement in a range of meaningful and personally challenging activities as an integral part of their lifestyle.

# 5. Are the elements clearly stated?

|  | Strongly Agree |
| --- | --- |
|  | Somewhat Agree |
|  | Neither Agree Nor Disagree |
|  | Somewhat Disagree |
|  | Strongly Disagree |

# 6. Do you agree with the elements?

|  | Strongly Agree |
| --- | --- |
|  | Somewhat Agree |
|  | Neither Agree Nor Disagree |
|  | Somewhat Disagree |
|  | Strongly Disagree |

# Core Principles

There are core principles implicit in the definition. Physical literacy: Is an inclusive concept accessible to all; Represents a unique journey for each individual; Should be cultivated and enjoyed through exposure to a range of different environments and contexts; and Should be valued and nurtured throughout life.

# 7. Are the core principles clearly stated?

|  | Strongly Agree |
| --- | --- |
|  | Somewhat Agree |
|  | Neither Agree Nor Disagree |
|  | Somewhat Disagree |
|  | Strongly Disagree |

# 8. Do you agree with the core principles?

|  | Strongly Agree |
| --- | --- |
|  | Somewhat Agree |
|  | Neither Agree Nor Disagree |
|  | Somewhat Disagree |
|  | Strongly Disagree |

#

Canada’s Physical Literacy Consensus Statement is the result of a collaborative process between ParticipACTION, Sport for Life Society, the Healthy Active Living and Obesity Research Group at the Children’s Hospital of Eastern Ontario Research Institute, PHE Canada, Canadian Parks and Recreation Association, and the Ontario Society of Physical Activity Promoters in Public Health. Representatives from the International Physical Literacy Association also contributed in an advisory capacity.

# 9. Is this Physical Literacy Consensus Statement important to you and/or your job?

|  | Yes |
| --- | --- |
|  | No |

# 10. Ignore this question. It is for French survey respondents only.

|  | -- |
| --- | --- |
|  | -- |
|  | -- |
|  | -- |
|  | -- |

# 11. Ignore this question. It is for French survey respondents only.

|  | -- |
| --- | --- |
|  | -- |
|  | -- |
|  | -- |
|  | -- |

# 12. In the box below, please enter any comments that you would like to add regarding this Physical Literacy Consensus Statement.

# 13. With what sector do you primarily associate?

|  | Sport sector |
| --- | --- |
|  | Education sector |
|  | Recreation sector |
|  | Childcare sector |
|  | Healthcare sector |
|  | Public health sector |
|  | Physical activity/fitness sector |
|  | Research sector |
|  | Government sector |
|  | Other, please specify... ______________________ |

# 14. Where do you primarily live / practice?

|  | Alberta |
| --- | --- |
|  | British Columbia |
|  | Manitoba |
|  | New Brunswick |
|  | Newfoundland and Labrador |
|  | Northwest Territories |
|  | Nova Scotia |
|  | Nunavut |
|  | Ontario |
|  | Prince Edward Island |
|  | Québec |
|  | Saskatchewan |
|  | Yukon Territory |
|  | Outside Canada |
|  | If you selected outside Canada, please indicate country... ______________________ |

# 15. When the final version of the Physical Literacy Consensus Statement is complete, would you like to be contacted for final review so that if supportive, you can decide if you would like to be listed in a “supported by” section associated with the Consensus Statement?

|  | Yes |
| --- | --- |
|  | No |
|  | Don't Know |

# 16. If you answered "Yes" to the previous question, please provide your email address so we can send you the final version and if supportive, gather your name, how you would like to be identified, and province/country to be listed in a “supported by” section associated with the Consensus Statement.

Déclaration de consensus canadien sur la littératie physique

#

**Titre de l’étude** : Sondage d’opinion de la Déclaration de consensus canadien sur la littératie physique 

**Chercheurs** :

Mark Tremblay (PhD), Groupe de recherche sur les saines habitudes de vie et l’obésité, Centre hospitalier pour enfants de l’est de l’Ontario (CHEO), courriel : [mtremblay@cheo.on.ca](mailto:mtremblay@cheo.on.ca)

Comme vous êtes un professionnel ou un chercheur dont le travail est relié à la littératie physique ou à la promotion de l’activité physique, du sport et des loisirs, nous vous invitons à participer à un sondage et à nous faire part de votre opinion sur le projet de consensus canadien sur la littératie physique.
 
Devant l’intérêt grandissant de divers intervenants pour la littératie physique, plusieurs développements récents ont permis de faire la promotion de ce concept à travers une foule d’initiatives. L’enthousiasme autour de l’apparition du concept a aussi suscité l’émergence d’un bon nombre de définitions, mais également à une utilisation parfois erronée de l’expression que l’on croit, à tort, pouvoir utiliser de façon interchangeable avec « activité physique », « éducation physique », « habiletés motrices de base » ou « développement des habiletés motrices ». Une consultation auprès des leaders canadiens dans le domaine a permis d’identifier que de rédiger une définition commune de la littératie physique était non seulement nécessaire mais aussi souhaitée. Une définition uniforme appuyée par un langage cohérent permettrait davantage de clarté pour le développement de politiques, de pratiques, et pour la recherche.
 
La déclaration consensuelle a été élaborée par un comité directeur composé de dix personnes provenant de sept organismes avec une portée canadienne et internationale*. Ces organismes, possédant des perspectives et définitions variées de la littératie physique, se sont réunis pour développer une déclaration consensuelle. Le comité en est venu à la décision de proposer la définition développée par l’International Physical Literacy Association, qui est le résultat de connaissances scientifiques et de consultations avec différents secteurs.
 
La déclaration consensuelle propose une définition de la littératie physique, ainsi qu’une description des composantes de la définition. À cette étape finale du processus, nous souhaitons obtenir des commentaires sur la clarté de la déclaration. Nous aimerions également évaluer le niveau d’accord ou de désaccord avec la déclaration, l’importance accordée à celle-ci et le soutien éventuel de nombreux professionnels représentés par chacun des collaborateurs (le sport, les loisirs, l’éducation, la santé publique, la promotion de la santé, l’activité physique, etc.).
 
L’accord sur une définition commune est crucial à la planification stratégique des politiques, de la pratique et de la recherche afin de promouvoir la littératie physique. À la suite de cette consultation auprès des intervenants et des révisions subséquentes, la déclaration consensuelle finale sera rendue publique à la Conférence sur la littératie physique de Vancouver, en Colombie-Britannique, Canada, en juin 2015. D’autres discussions mèneront à l’écriture et à la publication d’un document de référence plus détaillé sur le processus et les résultats en lien avec l’élaboration de ce consensus.
 
 
La participation à ce sondage est tout à fait volontaire. En accédant à ce sondage et en le remplissant, vous donnez votre consentement implicite/passif à y participer. Nous ne vous demanderons pas votre nom ni votre adresse courriel à travers ce sondage et les réponses ne seront présentées que sous une forme regroupée. Un résumé des résultats sera disponible sur le site Web de ParticipACTION (www.ParticipACTION.com) une fois publié et sera aussi envoyé aux organisations participantes pour distribution à leurs membres. Veuillez communiquer avec Mark Tremblay au 613 737-7600, poste 4114, ou au mtremblay@cheo.on.ca pour toute question au sujet de cette étude. Le comité d’éthique de la recherche (CER) du Centre hospitalier pour enfants de l’est de l’Ontario (CHEO) a révisé ce protocole. Le CER étudie les aspects éthiques de toute recherche qui implique les sujets humains au CHEO et à son institut de recherche. Si vous avez des questions au sujet de vos droits en tant que participant à l’étude, veuillez communiquer avec le président du CER du CHEO au 613 737-7600, poste 3624. Note : Cette déclaration consensuelle est présentée en version préliminaire et ne doit pas être distribuée. 
 
 
Pour obtenir plus de renseignements sur le sondage et des directives sur comment débuter, cliquez sur le lien ci-dessous. Merci de votre temps!
 
<http://fluidsurveys.com/s/physical-literacy/>
 
**Nous vous encourageons à partager ce courriel et le lien vers le sondage avec vos collègues et au sein de votre réseau. Ce sondage sera disponible jusqu’au 24 avril 2015.**
 
*La déclaration de consensus canadien sur la littératie physique résulte d'une collaboration entre ParticipACTION, la Société le sport c'est pour la vie, le Groupe de recherche sur les saines habitudes de vie et l'obésité de l’Institut de recherche du Centre hospitalier pour enfants de l’est de l’Ontario, EPS Canada, l'Association canadienne des parcs et loisirs et l’Ontario Society of Physical Activity Promoters in Public Health. Des représentants de l’International Physical Literacy Association ont également contribué à cet ouvrage à titre de conseillers.

# Déclaration de consensus canadien sur la littératie physique

Devant l’intérêt grandissant de divers intervenants pour la littératie physique, plusieurs développements récents ont permis de faire la promotion de ce concept à travers une foule d’initiatives. L’enthousiasme autour de l’apparition du concept a aussi suscité l’émergence d’un bon nombre de définitions, mais également à une utilisation parfois erronée de l’expression que l’on croit, à tort, pouvoir utiliser de façon interchangeable avec « activité physique », « éducation physique », « habiletés motrices de base » ou « développement des habiletés motrices ». Une consultation auprès des leaders canadiens dans le domaine a permis d’identifier que de rédiger une définition commune de la littératie physique était non seulement nécessaire mais aussi souhaitée. Une définition uniforme appuyée par un langage cohérent permettrait davantage de clarté pour le développement de politiques, de pratiques, et pour la recherche.
 
Cette déclaration vise à :

Promouvoir la valeur de la littératie physique et préserver l’intégrité du concept;

Recommander l’usage d’une définition commune de la littératie physique telle que définie par l’*International Physical Literacy Association*;

Faciliter un positionnement stratégique au sein même de tous les secteurs représentés de la communauté de la littératie physique et entre ces secteurs;

Proposer une orientation afin d’améliorer la cohérence et la clarté des communications en lien avec la littératie physique;

Soutenir le développement cohérent et coordonné d’outils et de ressources sur la littératie physique créés par les intervenants du secteur.

# 1. La nécessité du consensus est-elle clairement présentée?

|  | Totalement en accord |
| --- | --- |
|  | Passablement en accord |
|  | Ni en accord, ni en désaccord |
|  | Passablement en désaccord |
|  | Totalement en désaccord |

# 2. Êtes-vous d'accord avec l'utilité du consensus?

|  | Totalement en accord |  |
| --- | --- | --- |
|  | Passablement en accord |  |
|  | Ni en accord, ni en désaccord |  |
|  | Passablement en désaccord |  |
|  | Totalement en désaccord |  |

# Définition de la littératie physique

La littératie physique se définit par la motivation, la confiance, la compétence physique, le savoir et la compréhension qu’une personne possède et qui lui permettent de valoriser et de se responsabiliser vis-à-vis son engagement envers l’activité physique durant toute sa vie. - Traduit de l’International Physical Literacy Association

# 3. La définition du consensus est-elle clairement présentée?

|  |  | Totalement en accord |  |
| --- | --- | --- | --- |
|  |  | Passablement en accord |  |
|  |  | Ni en accord, ni en désaccord |  |
|  |  | Passablement en désaccord |  |
|  |  | Totalement en désaccord |  |

# 4. Êtes-vous d'accord avec la définition du consensus?

|  | Totalement en accord |  |
| --- | --- | --- |
|  | Passablement en accord |  |
|  | Ni en accord, ni en désaccord |  |
|  | Passablement en désaccord |  |
|  | Totalement en désaccord |  |

# Les composantes de la littératie physique

La définition de la littératie physique comprend quatre composantes, qui se recoupent et se chevauchent :
 
a. Affective – **« motivation et confiance »**
La motivation et la confiance réfèrent à l’enthousiasme, à l’émotion positive et à la confiance en soi suscités à l’idée d’intégrer l’activité physique à son mode de vie, ce qui est l’objectif fondamental des professionnels de la promotion de la littératie physique.
 
b. Cognitive – **« savoir et compréhension »**
Le savoir et la compréhension sous-entendent la capacité d’identifier et d’exprimer clairement les qualités essentielles qui influencent le mouvement, la compréhension des bénéfices santé du mode de vie actif et la conscience des éléments qui permettent la pratique sécuritaire de l’activité physique, et ce, dans un éventail de situations et d’environnements physiques différents.
 
c. Physique – **« compétence physique »**
La compétence physique réfère à la capacité d’une personne à développer des habitudes et habiletés motrices, et à expérimenter différentes intensités et durées de mouvements. Optimiser ses compétences physiques permet de participer à un large éventail de situations et d’activités physiques.
 
d. Comportementale – **« engagement dans l’activité physique pour la vie »**
L’engagement d’un individu dans des activités physiques pour la vie s’appuie sur sa responsabilisation à l’égard de la littératie physique, résultant par son libre choix d’être actif de façon régulière. Cet individu priorisera et maintiendra cette implication comme une partie importante de son mode de vie à travers une panoplie d’activités signifiantes et de défis personnels.

# 5. Les composantes de la littératie physique sont-elles clairement présentées?

|  | Totalement en accord |  |
| --- | --- | --- |
|  | Passablement en accord |  |
|  | Ni en accord, ni en désaccord |  |
|  | Passablement en désaccord |  |
|  | Totalement en désaccord |  |

# 6. Êtes-vous d'accord avec ces composantes?

|  | Totalement en accord |  |
| --- | --- | --- |
|  | Passablement en accord |  |
|  | Ni en accord, ni en désaccord |  |
|  | Passablement en désaccord |  |
|  | Totalement en désaccord |  |

# Principes fondamentaux

La définition proposée de littératie physique s’appuie de façon implicite sur les principes fondamentaux suivants. La littératie physique :

- est un concept inclusif accessible à tous;
- représente un parcours unique pour chaque individu;
- devrait être cultivée et appréciée au moyen d’une exposition à différents environnements et contextes; et
- devrait être valorisée et encouragée tout au long de la vie.

# 7. Les principes fondamentaux en lien avec la littératie physique sont-ils clairement présentés?

|  | Totalement en accord |  |
| --- | --- | --- |
|  | Passablement en accord |  |
|  | Ni en accord, ni en désaccord |  |
|  | Passablement en désaccord |  |
|  | Totalement en désaccord |  |

# 8. Êtes-vous d'accord avec ces principes fondamentaux?

|  |  | Totalement en accord |  |
| --- | --- | --- | --- |
|  |  | Passablement en accord |  |
|  |  | Ni en accord, ni en désaccord |  |
|  |  | Passablement en désaccord |  |
|  |  | Totalement en désaccord |  |

#

La déclaration de consensus canadien sur la littératie physique résulte d'une collaboration entre ParticipACTION, la Société le sport c'est pour la vie, le Groupe de recherche sur les saines habitudes de vie et l'obésité de l’Institut de recherche du Centre hospitalier pour enfants de l’est de l’Ontario, EPS Canada, l'Association canadienne des parcs et loisirs et l’Ontario Society of Physical Activity Promoters in Public Health. Des représentants de l’International Physical Literacy Association ont également contribué à cet ouvrage à titre de conseillers.

# 9. Le présent consensus au sujet de la littératie physique est-il important pour vous et/ou votre travail?

|  | Oui |
| --- | --- |
|  | Non |

# 10. Dans quelle mesure êtes-vous d'accord avec l'utilisation de l'expression "littératie physique" pour désigner en français le concept de "physical literacy"?

|  | Totalement en accord |
| --- | --- |
|  | Passablement en accord |
|  | Ni en accord, ni en désaccord |
|  | Passablement en désaccord |
|  | Totalement en désaccord |
|  |  |

# 11. Dans quelle mesure auriez-vous préféré que l'expression "savoir-faire physique" désigne en français le concept de "physical literacy"?

|  | Totalement en accord |
| --- | --- |
|  | Passablement en accord |
|  | Ni en accord, ni en désaccord |
|  | Passablement en désaccord |
|  | Totalement en désaccord |

# 12. S'il vous plaît, partagez-nous tout commentaire supplémentaire que vous souhaiteriez mentionner au sujet du présent Consensus canadien sur la littératie physique :

# 13. Dans quel domaine/secteur oeuvrez-vous principalement?

|  | Domaine du sport |
| --- | --- |
|  | Domaine de l'éducation |
|  | Domaine récréatif |
|  | Domaine du soin aux enfants |
|  | Domaine de la santé en général |
|  | Domaine de la santé publique |
|  | Domaine de l'activité physique |
|  | Domaine de la recherche |
|  | Secteur gouvernemental |
|  | Autre - précisez SVP :______________________ |

# 14. Dans quelle province ou territoire habitez / travaillez-vous?

|  | Alberta |
| --- | --- |
|  | Colombie-Britannique |
|  | Manitoba |
|  | Nouveau-Brunswick |
|  | Terre-Neuve-et-Labrador |
|  | Territoires du Nord-Ouest |
|  | Nouvelle-Écosse |
|  | Nunavut |
|  | Ontario |
|  | Île-du-Prince-Édouard |
|  | Québec |
|  | Saskatchewan |
|  | Yukon Territory |
|  | À l'extérieur du Canada |
|  | Si vous avez sélectionné « À l'extérieur du Canada », indiquez-nous dans quel pays :______________________ |

# 15. Lorsque la version finale du Consensus canadien sur la littératie physique sera établie, souhaiteriez-vous être contacté pour une révision finale? Dans un tel cas, vous pourriez alors apparaître au sein de la liste de collaborateurs ayant participé à l'élaboration du consensus (selon votre discrétion).

|  | Oui |
| --- | --- |
|  | Non |
|  | Je ne sais pas |

# 16. Si vous avez répondu « Oui » à l'énoncé précédent, veuillez s'il vous plaît nous indiquer l'adresse courriel à laquelle vous souhaiteriez que la version finale du consensus vous parvienne. De plus, si vous souhaitez apparaitre au sein de la liste de collaborateurs ayant participé à l'élaboration du consensus, complétez les informations subséquentes :
