# Supplementary material for: Canada’s Physical Literacy Consensus Statement: process and outcome
Source: BMC Public Health. 2018 Oct 2;18(Suppl 2):1034. doi: 10.1186/s12889-018-5903-x (PMC6167775; doi:10.1186/s12889-018-5903-x)
Supplement: Supplementary file 3 — Detailed results from the Stakeholder Survey. (DOCX 50 kb) [file 12889_2018_5903_MOESM3_ESM.docx]

Additional File 3. Detailed results from the Stakeholder Survey.

Table S1. Stakeholder assessment of the Consensus Statement’s purpose (questions 1 and 2), by location.

|  | Purpose clearly stated | | | | Agreement with purpose | | | |
| --- | --- | --- | --- | --- | --- | --- | --- | --- |
| Location | Total N | Somewhat agree | Strongly agree | Combined agreement | Total N | Somewhat agree | Strongly agree | Combined agreement |
| **All respondents***  All locations  East  Ontario / Québec  West/north  International | **1374**  1307  130  536  588  53 | **444 (32.3%)**  419 (32.1%)  42  (32.3%)  156 (29.1%)  209 (35.5%)  12  (22.6%) | **847 (61.6%)**  814 (62.2%)  86 (66.2%)  344 (64.2%)  344 (58.5%)  40 (75.5%) | **1291 (93.9%)**  1233 (94.3%)  128 (98.5%)  500 (93.3%)  553 (94.0%)  52  (98.1%) | **1369**  1303  130  537  583  53 | **358 (26.2%)**  340 (26.1%)  29  (22.3%)  133 (24.8%)  169 (29.0%)  9  (17.0%) | **947 (69.2%)**  904 (69.4%)  96 (73.9%)  372 (69.2%)  393 (67.4%)  43 (81.1%) | **1305 (95.4%)**  1244 (95.5%)  125 (96.2%)  505  (94.0)  562 (96.4%)  52  (98.1%) |

* “All respondents” includes those who answered the question but did not identify a location.

Table S2. Stakeholder assessment of the Consensus Statement’s purpose (questions 1 and 2), by sector.

|  | Purpose clearly stated | | | | Agreement with purpose | | | |
| --- | --- | --- | --- | --- | --- | --- | --- | --- |
| Location | Total N | Somewhat agree | Strongly agree | Combined agreement | Total N | Somewhat agree | Strongly agree | Combined agreement |
| **All respondents***  All sectors  Childcare/education  Government  Healthcare/public health  Physical activity/fitness/  recreation/sport  Research  Other | **1374**  1311  402  54  166  537  62  90 | **444 (32.3%)**  421 (32.1%)  139 (34.6%)  18 (33.3%)  50 (30.1%)  173 (32.2%)  17 (27.4%)  24 (26.7%) | **847 (61.6%)**  815 (62.2%)  242 (60.2%)  33 (61.1%)  112 (67.5%)  325 (60.5%)  44 (71.0%)  59 (65.5%) | **1291 (93.9%)**  1236 (94.3%)  381 (94.8%)  51  (94.4%)  162 (97.6%)  498 (92.7%)  61  (98.4%)  83  (92.2%) | **1369**  1306  401  54  166  534  62  89 | **358 (26.2%)**  342 (26.2%)  114 (28.4%)  12 (22.2%)  39 (23.5%)  145 (27.2%)  11 (17.7%)  21 (23.6%) | **947 (69.2%)**  905 (69.3%)  272 (67.9%)  38 (70.4%)  122 (73.5%)  362 (67.7%)  48 (77.5%)  63 (70.8%) | **1305 (95.4%)**  1247 (95.5%)  386 (96.3%)  50  (92.6%)  161 (97.0%)  507 (94.9%)  59  (95.2%)  84  (94.4%) |

* All respondents include those who answered the question but did not identify a sector.

Table S3. Stakeholder assessment of the Consensus Statement’s definition (questions 3 and 4), by location.

|  | Definition clearly stated | | | | Agreement with definition | | | |
| --- | --- | --- | --- | --- | --- | --- | --- | --- |
| Location | Total N | Somewhat agree | Strongly agree | Combined agreement | Total N | Somewhat agree | Strongly agree | Combined agreement |
| **All respondents***  All locations  East  Ontario / Québec  West/North  International | **1370**  1306  130  536  587  53 | **480**  **(35%)**  459 (35.2%)  47  (36.2%)  189 (35.3%)  203 (34.6%)  20  (37.7%) | **742 (54.2%)**  713 (54.5%)  77 (59.2%)  283 (52.8%)  322 (54.8%)  31 (58.5%) | **1222 (89.2%)**  1172 (89.7%)  124 (95.4%) +  472 (88.1%) -  525 (89.4%)  51  (96.2%) + | **1366**  1302  130  533  586  53 | **482 (35.3%)**  456 (35.0%)  48  (36.9%)  181 (34.0%)  209 (35.7%)  18  (34.0%) | **708 (51.8%)**  685 (52.6%)  74 (56.9%)  265 (49.7%)  313 (53.4%)  33 (62.2%) | **1190 (87.1%)**  1141 (87.6%)  122  (93.8%) ++  446  (83.7%) --  522  (89.1%) ++  51  (96.2%) ++ |

* All respondents include those who answered the question but did not identify a location.

+ Slightly higher combined agreement than expected (Fischer’s exact test, *p* = .0294)

- Slightly lower combined agreement than expected (Fischer’s exact test, *p* = .0294)

++ Slightly higher combined agreement than expected (Fischer’s exact test, *p*< .001)

-- Slightly lower combined agreement than expected (Fischer’s exact test, *p*< .001)

Table S4. Stakeholder assessment of the Consensus Statement’s definition (questions 3 and 4), by sector.

|  | Definition clearly stated | | | | Agreement with definition | | | |
| --- | --- | --- | --- | --- | --- | --- | --- | --- |
| Location | Total N | Somewhat agree | Strongly agree | Combined agreement | Total N | Somewhat agree | Strongly agree | Combined agreement |
| **All respondents***  All sectors  Childcare/education  Government  Healthcare/public health  Physical activity/fitness/  recreation/sport  Research  Other | **1370**  1310  402  55  167  535  62  89 | **480 (35.0%)**  460 (35.1%)  127 (31.6%)  20 (36.4%)  64 (38.3%)  189 (35.3%)  26 (41.9%)  34 (38.2%) | **742 (54.2%)**  715 (54.6%)  243 (60.4%)  26 (47.2%)  87 (52.1%)  282 (52.7%)  30 (48.4%)  47 (52.8%) | **1222 (89.2%)**  1175 (89.7%)  370 (92.0%)  46  (83.6%)  151 (90.4%)  471 (88.0%)  56  (90.3%)  81  (91.0%) | **1366**  1306  399  55  167  535  62  88 | **482 (35.3%)**  459 (35.1%)  137 (34.3%)  16 (29.1%)  65 (38.9%)  192 (35.9%)  21 (33.9%)  28 (31.8%) | **708 (51.8%)**  685 (52.5%)  228 (57.1%)  25 (45.5%)  82 (49.1%)  270 (50.5%)  31 (50.0%)  49 (55.7%) | **1190 (87.1%)**  1144 (87.6%)  365  (91.5%) ++  41  (74.5%) --  147  (88.0%)  462  (86.4%)  52  (83.9%)  77  (87.5%) |

*All respondent includes those who answered the question but did not identify a sector.

++ Slightly higher combined agreement than expected (Fischer’s exact test, *p*< .01)

-- Slightly lower combined agreement than expected (Fischer’s exact test, *p*< .01)

Table S5. Stakeholder assessment of the Consensus Statement’s elements (questions 5 and 6), by location.

|  | Elements clearly stated | | | | Agreement with elements | | | |
| --- | --- | --- | --- | --- | --- | --- | --- | --- |
| Location | Total N | Somewhat agree | Strongly agree | Combined agreement | Total N | Somewhat agree | Strongly agree | Combined agreement |
| **All respondents***  All locations  East  Ontario / Québec  West/North  International | **1368**  1309  130  539  587  53 | **336 (24.6%)**  314 (24.0%)  25  (19.2%)  128 (23.7%)  147 (25.0%)  14  (26.4%) | **961 (70.2%)**  931 (71.1%)  100 (77.0%)  374 (69.4%)  419 (71.4%)  38 (71.7%) | **1297 (94.8%)**  1245 (95.1%)  125 (96.2%)  502 (93.1%)  566 (96.4%)  52  (98.1%) | **1365**  1303  130  534  586  53 | **330 (24.2%)**  309 (23.7%)  23  (17.7%)  123 (23.0%)  148 (25.3%)  15  (28.3%) | **927 (67.9%)**  898 (68.9%)  99 (76.1%)  358 (67.1%)  406 (69.2%)  35 (66.0%) | **1257 (92.1%)**  1207 (92.6%)  122  (93.8%)  481  (90.1%) --  554  (94.5%) ++  50  (94.3%) |

* All respondents include those who answered the question but did not identify a location.

++ Slightly higher combined agreement than expected (Fischer’s exact test, *p* = .03717)

-- Slightly lower combined agreement than expected (Fischer’s exact test, *p* = .03717)

Table S6. Stakeholder assessment of the Consensus Statement’s elements (questions 5 and 6), by sector.

|  | Elements clearly stated | | | | Agreement with elements | | | |
| --- | --- | --- | --- | --- | --- | --- | --- | --- |
| Location | Total N | Somewhat agree | Strongly agree | Combined agreement | Total N | Somewhat agree | Strongly agree | Combined agreement |
| **All respondents***  All sectors  Childcare/education  Government  Healthcare/public health  Physical activity/fitness/  recreation/sport  Research  Other | **1368**  1312  403  55  167  535  62  90 | **336 (24.6%)**  317 (24.1%)  74 (18.4%)  22 (40.0%)  45 (26.9%)  132 (24.7%)  20 (32.3%)  24 (26.7%) | **961 (70.2%)**  931 (71.0%)  312 (77.4%)  32 (58.2%)  116 (69.5%)  368 (68.8%)  40 (64.5%)  63 (70.0%) | **1297 (94.8%)**  1248 (95.1%)  386 (95.8%)  54  (98.2%)  161 (96.4%)  500 (93.5%)  60  (96.8%)  87  (96.7%) | **1365**  1307  400  54  166  535  62  90 | **330 (24.2%)**  311 (23.8%)  86 (21.5%)  17 (31.5%)  43 (25.9%)  129 (24.1%)  14 (22.6%)  22 (24.4%) | **927 (67.9%)**  899 (68.8%)  292 (73.0%)  31 (57.4%)  115 (69.3%)  359 (67.1%)  41 (66.1%)  61 (67.8%) | **1257 (92.1%)**  1210 (92.6%)  378 (94.5%)  48  (88.9%)  158 (95.2%)  488 (91.2%)  55  (88.7%)  83  (92.2%) |

* All respondents include those who answered the question but did not identify a sector.

Table S7. Stakeholder assessment of the Consensus Statement’s principles (questions 7 and 8), by location.

|  | Principles clearly stated | | | | Agreement with principles | | | |
| --- | --- | --- | --- | --- | --- | --- | --- | --- |
| Location | Total N | Somewhat agree | Strongly agree | Combined agreement | Total N | Somewhat agree | Strongly agree | Combined agreement |
| **All respondents***  All locations  East  Ontario / Québec  West/North  International | **1332**  1306  129  538  587  52 | **280 (21.0%)**  272 (20.8%)  28 (21.7%)  121 (22.5%)  113 (19.2%)  12 (23.1%) | **996 (74.8%)**  979 (75.1%)  98 (76.0%)  401 (74.5%)  440 (75.0%)  40 (76.9%) | **1276 (95.8%)**  1253 (95.9%)  126 (97.7%)  522 (97.0%)  553 (94.2%) -  52 (100.0%) | **1333**  1306  130  538  585  53 | **247 (18.5%)**  235 (18.0%)  22 (16.9%)  100 (18.6%)  105 (17.9%)  8  (15.1%) | **1032 (77.4%)**  1019 (78.0%)  103 (79.3%)  416 (77.3%)  455 (77.8%)  45 (84.9%) | **1279 (95.9%)**  1254 (96.0%)  125 (96.2%)  516 (95.9%)  560 (95.7%)  53 (100.0%) |

* All respondents include those who answered the question but did not identify a location.

- Slightly lower combined agreement than expected (Fischer’s exact test, *p* = .03395)

Table S8. Stakeholder assessment of the Consensus Statement’s principles (questions 7 and 8), by sector.

|  | Principles clearly stated | | | | Agreement with principles | | | |
| --- | --- | --- | --- | --- | --- | --- | --- | --- |
| Location | Total N | Somewhat agree | Strongly agree | Combined agreement | Total N | Somewhat agree | Strongly agree | Combined agreement |
| **All respondents***  All sectors  Childcare/education  Government  Healthcare/public health  Physical activity/fitness/  recreation/sport  Research  Other | **1332**  1309  401  55  167  534  62  90 | **280 (21.0%)**  275 (21.0%)  75 (18.7%)  15 (27.3%)  34 (20.4%)  122 (22.8%)  8  (12.9%)  21 (23.3%) | **996 (74.8%)**  980 (74.9%)  312 (77.8%)  36 (65.4%)  129 (77.2%)  384 (72.0%)  52 (83.9%)  67 (74.5%) | **1276 (95.8%)**  1255 (95.9%)  387 (96.5%)  51  (92.7%)  163 (97.6%)  506 (94.8%)  60  (96.8%)  88  (97.8%) | **1333**  1310  403  55  166  534  62  90 | **247 (18.5%)**  239 (18.2%)  68 (16.9%)  15 (27.3%)  35 (21.1%)  94 (17.6%)  7  (11.3%)  17 (18.9%) | **1032 (77.4%)**  1019 (77.8%)  319 (79.1%)  37 (67.2%)  130 (78.3%)  412 (77.2%)  55 (88.7%)  70 (77.8%) | **1279 (95.9%)**  1258 (96.0%)  387 (96.0%)  52  (94.5%)  165 (99.4%)  506 (94.8%)  61  (98.4%)  87  (96.7%) |

* All respondents include those who answered the question but did not identify a sector.
